# Lactucopicrin promotes the autophagic degradation of MAP2K4/MKK4 by mediating CCDC50 palmitoylation to alleviate osteoarthritis progression

**DOI:** 10.1080/15548627.2025.2601041

**Published:** 2026-01-21

**Authors:** Wenjun Li, Qijie Sun, Konghe Hu, Dongmei Tang, Cheng Yang, Yingchao Xie, Xiaodong Peng, Yongtao Deng, Jiansen Lu, Yong Qi, Yifen Lin, Hongtao Sun, Qinyu Tian, Changpeng Xu, Xinggui Tian, Huaji Jiang

**Affiliations:** a Department of Orthopedics, The Affiliated Guangdong Second Provincial General Hospital of Jinan University, Guangzhou, China; b Department of Orthopaedics, The State Key Clinical Specialty in Orthopaedics, Yuebei People’s Hospital, Affiliated to Shantou University Medical College, Shaoguan, China; c Department of Orthopedics, Xinzhou People’s Hospital, Xinzhou, China; d Department of Urology, The Third Affiliated Hospital of Southern Medical University, Guangzhou, China; e Department of Immunology, School of Basic Medical Sciences, Southern Medical University, Guangzhou, China; f Department of Joint Surgery, Shaoguan First People’s Hospital, Southern Medical University, Shaoguan, China; g Department of Orthopaedics and Traumatology, Faculty of Medicine, The Chinese University of Hong Kong, Hong Kong SAR, China; h University Center of Orthopaedic, Trauma and Plastic Surgery, University Hospital Carl Gustav Carus at TUD Dresden University of Technology, Dresden, Germany; i Center for Translational Bone, Joint and Soft Tissue Research, University Hospital Carl Gustav Carus at TUD Dresden University of Technology, Dresden, Germany; j Center for Orthopaedics, Trauma Surgery and Rehabilitation Medicine, University Medicine Greifswald, Greifswald, Germany

**Keywords:** Autophagy, CCDC50, chondrocyte senescence, MAP2K4/MKK4, osteoarthritis, palmitoylation

## Abstract

Macroautophagy/autophagy plays a crucial role in maintaining cellular homeostasis and protecting against osteoarthritis (OA). Its dysregulation contributes to OA progression by promoting chondrocyte senescence, inflammation, and cartilage degradation. Enhancing autophagic activity thus represents a promising therapeutic strategy for OA. In this study, we identified lactucopicrin (LCP) as an effective autophagy activator that alleviates OA progression in a mouse model induced by the destabilization of the medial meniscus, by reducing cartilage degeneration and preserving matrix integrity. Mechanistically, LCP enhances ZDHHC4-catalyzed palmitoylation of the cargo receptor CCDC50, facilitating the selective autophagic degradation of MAP2K4/MKK4, leading to the suppression of MAPK/JNK signaling and the attenuation of chondrocyte senescence. Structural analysis reveals that LCP directly binds to His72 of ZDHHC4 *via* its p-hydroxybenzoic acid moiety, boosting enzymatic activity and promoting selective autophagy. These findings establish a novel ZDHHC4-CCDC50-MAP2K4/MKK4-MAPK/JNK regulatory axis linking palmitoylation, autophagy, and senescence, and identify LCP as a promising agent for targeting this pathway to inhibit OA progression. Furthermore, this study provides mechanistic insights into the crosstalk between autophagy, protein palmitoylation, and cellular senescence in degenerative joint disease.

**Abbreviation**: ABE: acyl-biotin exchange; ADAMTS5: ADAM metallopeptidase with thrombospondin type 1 motif 5; CCDC50: coiled-coil domain containing 50; COL2A1: collagen, type II, alpha 1; COL10A1: collagen, type X, alpha 1; DARTS: drug affinity responsive target stability; DHHC: Asp-His-His-Cys catalytic motif; GOT1/AST: glutamic-oxaloacetic transaminase 1, soluble; GPT/ALT: glutamic pyruvic transaminase, soluble; H_2_O_2:_ hydrogen peroxide; LCP: lactucopicrin; IL6: interleukin 6; MAPK/JNK: mitogen-activated protein kinase; MAP2K4/MKK4: mitogen-activated protein kinase kinase 4; MMP13: matrix metallopeptidase 13; OA: osteoarthritis; p-MAPK/JNK: phosphorylated mitogen-activated protein kinase; SASP: senescence-associated secretory phenotype; SA-GLB1/β-gal: senescence-associated galactosidase, beta 1; ZDHHC: zinc finger, DHHC domain containing.

## Introduction

Macroautophagy/Autophagy, a highly conserved cellular degradation and recycling process, is essential for maintaining cellular homeostasis by selectively eliminating damaged proteins, organelles, and invading pathogens [1,2]. Dysregulation of autophagy contributes to the pathogenesis of various degenerative diseases, including osteoarthritis (OA) [1,2]. In healthy chondrocytes, autophagy plays a protective role by clearing damaged organelles and misfolded proteins, thus preserving cartilage integrity and preventing OA onset [3,4]. However, in aging or OA-affected chondrocytes, autophagy exhibits both protective and potentially cytotoxic effects [3,4]. Although autophagy modulation presents a promising therapeutic strategy for OA [1,2], the molecular mechanisms underlying its contribution to OA progression remain largely unexplored.

Chondrocyte senescence, driven by aging, joint trauma, or chronic stress, is increasingly recognized as a central contributor to OA pathogenesis [5,6]. Senescent chondrocytes exhibit a senescence-associated secretory phenotype (SASP), characterized by the secretion of pro-inflammatory chemokines, cytokines, proteases, and growth factors, which exacerbate joint degeneration and destruction [5,6]. Given the detrimental impact of cellular senescence on joint integrity, targeting chondrocyte senescence-associated pathways holds substantial therapeutic potential for OA [5,6].

Sustained activation of the MAPK/JNK (mitogen-activated protein kinase) pathway promotes OA progression by amplifying stress signals, matrix degradation, and inflammatory responses [7]. Consequently, the MAPK/JNK pathway has been identified as a promising therapeutic target for OA [7]. The MAPK/JNK pathway is mediated by upstream kinase kinases (MAP2Ks), especially MAP2K4/MKK4 (mitogen-activated protein kinase kinase 4) and MAP2K7/MKK7 (mitogen-activated protein kinase kinase 7) [8]. Recent research has reported that mechanical overload activates the MAP2K7-MAPK/JNK axis, promoting chondrocyte senescence and cartilage degradation [9]. However, the specific role of MAP2K4/MKK4 in modulating MAPK/JNK-driven chondrocyte senescence and OA progression remains unclear. Furthermore, MAPK/JNK signaling is also involved in autophagic processes induced by various stimuli [10], suggesting that it may function as a critical link between autophagy and chondrocyte senescence in OA pathogenesis. Therefore, enhancing autophagy to suppress the MAP2K4/MKK4-MAP2K7/MKK7-MAPK/JNK signaling axis may represent a promising strategy to delay chondrocyte senescence and slow OA development.

Selective autophagy relies on cargo receptors that recognize specific substrates for targeted degradation [11]. Among these, CCDC50 (coiled-coil domain containing 50) has recently been proposed as a novel autophagy receptor involved in various cellular processes, including growth factor signaling, inflammation, apoptosis, and cell death [12]. Despite its diverse functions, its role in selective autophagy and OA pathogenesis has not been elucidated, and the molecular mechanisms that regulate CCDC50 activity are still poorly understood.

Protein palmitoylation involves the reversible attachment of fatty acid chains to cysteine residues on proteins through thioester bonds [13,14]. This dynamic modification regulates essential cellular processes, including protein stability, subcellular localization, membrane trafficking, protein-protein interactions, and enzymatic activity. Palmitoylation is catalyzed by a family of protein acyltransferases (PATs) containing a conserved Asp-His-His-Cys (DHHC) motif [13,14]. In humans and mice, 23 ZDHHC (zinc finger, DHHC domain containing) isoforms have been identified, each exhibiting distinct substrate specificities and catalytic efficiencies [13,14]. Dysregulation of PAT activity and aberrant palmitoylation have been implicated in multiple diseases, including cancer, neurodegeneration, inflammation, and immune disorders, underscoring the therapeutic potential of targeting ZDHHC enzymes [13]. However, the role of palmitoylation in OA progression, particularly in the context of selective autophagy, remains largely unexplored.

Lactucopicrin (LCP), a sesquiterpene lactone primarily extracted from *Lactuca virosa* (wild lettuce) and *Cichorium intybus* (chicory) [15], exhibits multiple pharmacological properties, including anti-inflammatory [16], analgesic [17], and neurite outgrowth-promoting effects [15]. While several natural compounds have been reported to alleviate OA progression by modulating autophagy in chondrocytes [18], the specific role of LCP in this context remains unclear. Recent findings suggest that LCP may activate autophagy and reduce chronic inflammation [19], making it a promising candidate for OA intervention.

Based on these findings, we hypothesize that LCP promotes CCDC50 palmitoylation, thereby facilitating the selective autophagic degradation of MAP2K4/MKK4 and inhibiting MAPK/JNK-mediated chondrocyte senescence and OA progression. This study aims to elucidate the molecular mechanism by which LCP regulates selective autophagy through the ZDHHC-CCDC50 axis, providing novel insights into autophagy-mediated regulation of MAPK/JNK signaling in chondrocyte senescence and highlighting its therapeutic potential in OA.

## Results

### LCP inhibits OA progression and cartilage degeneration in mice

**Figure 1.**
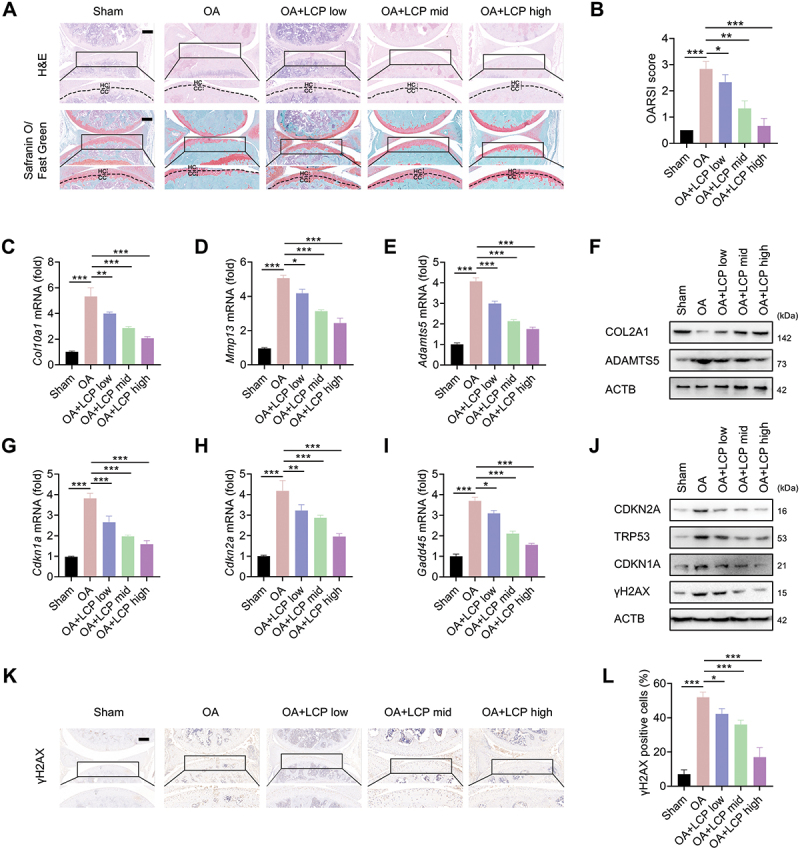
*Lactucopicrin (LCP) inhibits OA progression and cartilage degeneration in mice*. (**A**) Representative images of mouse articular cartilage stained with H&E, Safranin O/Fast Green; CC: calcified cartilage, and HC: hyaline cartilage. (**B**) Quantification of OA severity using the OARSI histological scoring system [20]. (**C–E**) qRT-PCR analysis of core OA molecular markers: *Col10a1/ColX* (collagen, type x, alpha 1), *Mmp13*, and *Adamts5*. (**F**) WB analysis of OA markers: COL2A1 (collagen type ii, alpha 1) and ADAMTS5. (**G–I**) qRT-PCR detection of senescence-associated genes: *Cdkn1a/p21, Cdkn2a/p16*^*INK4a*^, *and Gadd45*. (**J**) WB analysis of senescence-related proteins: CDKN2A/p16^INK4a^, CDKN1A/p21, TRP53/p53, and γH2AX. (**K-L**) Immunohistochemical staining of γH2AX in cartilage tissue and quantification of γH2AX-positive cells (%). Scale bar: 100 µm. Data are presented as mean ± SD. *p < 0.05, **p < 0.01, ***p < 0.001.

The chemical structure of LCP is shown in **Figure S1A**. To evaluate its therapeutic effects on OA, an OA mouse model was established *via* the destabilization of the medial meniscus (DMM) method. Histological staining revealed typical OA features in the untreated (OA) group, including cartilage thinning, decreased proteoglycans, disrupted cartilage architecture, fewer chondrocytes with irregular arrangements, and formation of chondrocyte “empty nests”. In contrast, LCP treatment effectively mitigated these pathological changes in a dose-dependent manner, with the high-dose group exhibiting cartilage morphology comparable to the sham group (Figure 1A). The severity of OA was quantified using the Osteoarthritis Research Society International (OARSI) scoring system [20], evaluating surface integrity, chondrocyte organization, and matrix staining, confirming its protective effect on cartilage (Figure 1B).

Molecular analyses further supported these observations. qRT-PCR assays demonstrated that LCP downregulated OA-related catabolic genes, including *Col10a1* (collagen, type X, alpha 1), *Mmp13* (matrix metallopeptidase 13), and *Adamts5* (ADAM metallopeptidase with thrombospondin type 1 motif 5) (Figure 1C–E). Western blot (WB) analysis revealed elevated protein levels of COL2A1 (collagen type II, alpha 1) and reduced ADAMTS5 in LCP-treated groups (Figure 1F). Furthermore, LCP also downregulated key markers of cartilage degeneration. qRT-PCR analyses revealed decreased expression of *Cdkn2a/p16*^*INK4a*^ (cyclin dependent kinase inhibitor 2A), *Cdkn1a/p21* (cyclin dependent kinase inhibitor 1A), and *Gadd45* (growth arrest and DNA-damage-inducible 45) (Figure 1G–I), which was further corroborated by WB results showing reduced levels of CDKN2A/p16^INK4a^, CDKN1A/p21, TRP53/p53 (transformation related protein 53), and γH2AX (Ser139 phosphorylated H2A.X variant histone; a DNA damage marker) (Figure 1J). Immunohistochemical (IHC) analysis confirmed the dose-dependent reduction of γH2AX expression in cartilage tissue (Figure 1K–L).

Importantly, LCP exhibited a favorable safety profile, as evidenced by the absence of significant changes in body weight or serum biochemical parameters, including GOT1/AST (glutamic-oxaloacetic transaminase 1, soluble), GPT/ALT (glutamic pyruvic transaminase, soluble), urea, and creatinine, in the high-dose treatment group compared to controls (**Figure S1B-F**). Pharmacokinetic analysis using liquid chromatography-tandem mass spectrometry (LC-MS/MS) revealed that LCP was rapidly distributed to joint tissues, reaching peak concentrations in cartilage 2 h after intraperitoneal injection and remaining detectable for up to 24 h (**Figure S1G-I**). These results confirm the effective intra-articular distribution and sustained retention of LCP following intraperitoneal injection. Collectively, these data demonstrate that LCP effectively attenuates OA progression and cartilage degeneration *in vivo* without inducing systemic toxicity.

### LCP inhibits chondrocyte senescence in vitro

**Figure 2.**
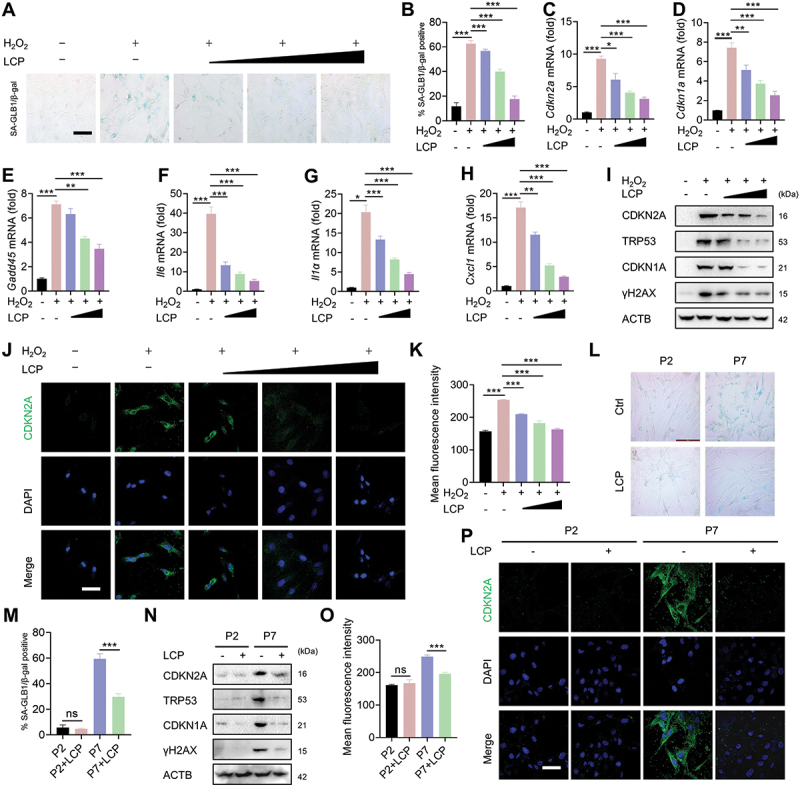
*LCP inhibits chondrocyte senescence in vitro*. (**A-K**) In the exogenous oxidative stress model induced by hydrogen peroxide (H_2_O_2_), LCP treatment inhibited chondrocyte senescence in a dose-dependent manner, as evidenced by (**A-B**) SA-GLB1/β-gal staining and corresponding quantification, (**C-H**) downregulation of senescence-associated markers (*Cdkn2a/p16, Cdkn1a/p21, Gadd45*) and SASP-related genes (*Il6, Il1α, Cxcl1*) as detected by qRT-PCR, (**I**) decreased protein levels of CDKN2A/p16^INK4a^, CDKN1A/p21, TRP53/p53, and γH2AX detected *via* WB, and (**J-K**) reduced CDKN2A/p16^INK4a^ expression shown by immunofluorescence staining and quantified fluorescence intensity. (**L-P**) In the natural senescence model induced by long-term *in vitro* culture (passage 7, P7), LCP also suppressed chondrocyte senescence, as demonstrated by: (**L-M**) SA-GLB1/β-gal staining and corresponding quantification, (**N**) reduced protein levels of CDKN2A/p16^INK4a^, CDKN1A/p21, TRP53/p53, and γH2AX in WB analysis, and (**O-P**) decreased CDKN2A/p16^INK4a^ expression in immunofluorescence staining and its quantification. Scale bar: 100 µm. Data are expressed as the mean ± SD. * *p* < 0.05, ** *p* < 0.01, *** *p* < 0.001. ns = not significant.

The anti-senescent effects of LCP were evaluated using two complementary *in vitro* models: an exogenous oxidative stress model induced by hydrogen peroxide (H_2_O_2_) and a natural senescence model through long-term culture (passage 7, P7) [21]. Cell Counting Kit-8 (CCK-8) assays confirmed that LCP was nontoxic within the therapeutic concentration range of 0–20 μM, which was used for subsequent experiments **(Figure S2A)**. In the H_2_O_2_-induced senescence model, LCP treatment significantly mitigated chondrocyte senescence in a dose-dependent manner, as demonstrated by the reduced expression of senescence-associated GLB1/galactosidase, beta (SA-GLB1/β-gal) by staining (Figure 2A,B), downregulation of senescence-associated genes, including cellular senescence markers (*Cdkn2a/p16, Cdkn1a/p21, Gadd45*) and SASP markers [*Il6* (interleukin 6), *Il1a* (interleukin 1 alpha), *Cxcl1* (C-X-C motif chemokine ligand 1)], as detected by qRT-PCR (Figure 2C–H). WB analysis further confirmed reduced protein levels of CDKN2A/p16^INK4a^, CDKN1A/p21, TRP53/p53, and γH2AX (Figure 2I). Consistently, immunofluorescence staining revealed a clear decline in CDKN2A/p16^INK4a^ expression (Figure 2J,K). In the long-term culture-induced natural senescence model (P7), LCP treatment consistently suppressed chondrocyte senescence, as evidenced by SA-GLB1/β-gal staining (Figure 2L,M) and downregulation of senescence-associated genes (*Cdkn2a/p16, Cdkn1a/p21, Gadd45*) and inflammatory SASP factors (*Il6, Il1a, Cxcl1*) as assessed by qRT-PCR analysis **(Figure S2B-G)**. WB analysis revealed reduced protein levels of CDKN2A/p16^INK4a^, CDKN1A/p21, TRP53/p53, and γH2AX (Figure 2N). Immunofluorescence staining further confirmed that LCP significantly reduced CDKN2A/p16^INK4a^ expression (Figure 2O,P).

Additionally, LCP was shown to effectively inhibit chondrocyte senescence induced by bleomycin, a known DNA-damaging agent. WB analysis revealed reduced expression of CDKN2A/p16^INK4a^, CDKN1A/p21, TRP53/p53, and γH2AX following LCP treatment **(Figure S2H)**. This anti-senescent effect was further validated in primary human chondrocytes isolated from patients who underwent unicompartmental knee arthroplasty. LCP treatment significantly suppressed senescence in these cells **(Figure S2I-Q)**. Together, these findings indicate that LCP effectively suppresses chondrocyte senescence in both murine and human chondrocytes, highlighting its potential as a therapeutic agent for targeting chondrocyte senescence in OA.

### LCP inhibits chondrocyte senescence via the MAPK/JNK signaling pathway

**Figure 3.**
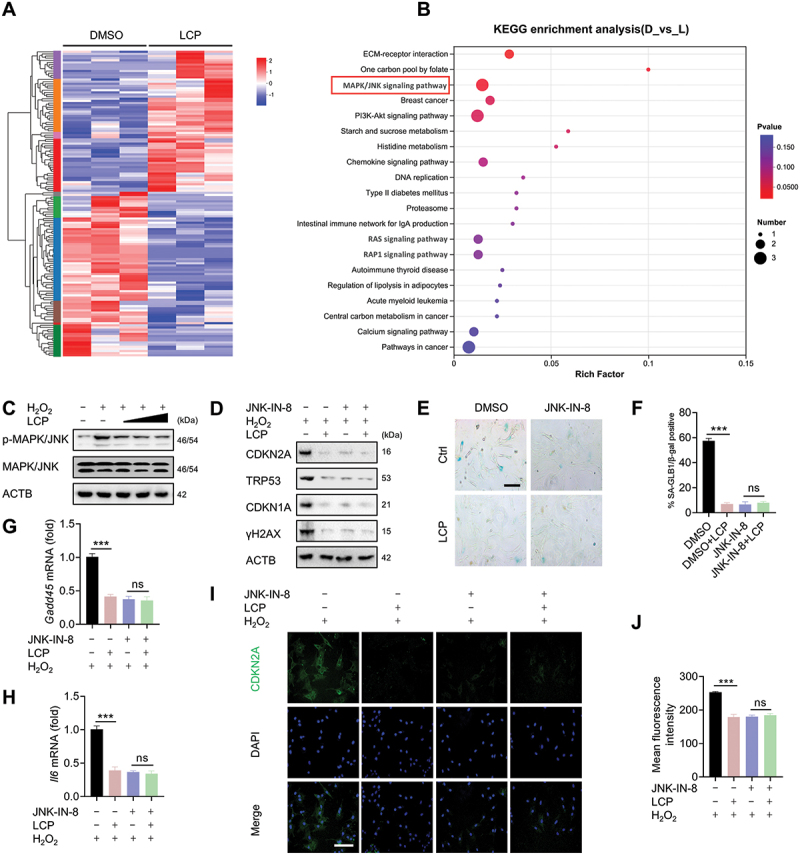
*LCP inhibits chondrocyte senescence via suppression of the MAPK/JNK signaling pathway*. (**A**) Heatmap showing hierarchical clustering of differentially expressed genes in chondrocytes treated with DMSO or LCP. (**B**) KEGG pathway enrichment analysis of differentially expressed genes (DEGs) identified from RNA-seq, indicating that the *MAPK/JNK* signaling pathway was significantly enriched and is highlighted in red, suggesting its potential involvement in the anti-senescent effects of LCP. (**C**) WB analysis showing reduced levels of phosphorylated MAPK/JNK (p-MAPK/JNK) following LCP treatment in H_2_O_2_-induced senescent chondrocytes, with total MAPK/JNK and ACTB/β-actin as loading controls. (**D**) WB analysis of senescence-associated markers (CDKN2A/p16^INK4a^, CDKN1A/p21, TRP53/p53, and γH2AX) in H_2_O_2_-induced chondrocytes, with or without LCP and/or the MAPK/JNK inhibitor JNK-IN-8 (2 μM). (**E-F**) Representative images of SA-GLB1/β-gal staining and corresponding quantification of SA-GLB1/β-gal-positive cells. (**G-H**) qRT-PCR analysis of senescence-related genes *Gadd45* and *Il6* showing reduced expression in response to LCP or JNK-IN-8 (2 μM) treatment. (**I-J**) Immunofluorescence staining of CDKN2A/p16^INK4a^ in H_2_O_2_-treated chondrocytes and corresponding quantification of fluorescence intensity. Scale bar: 100 µm. Data are expressed as mean ± SD. *** *p* < 0.001. ns = not significant.

To explore the signaling mechanisms underlying the anti-senescent effects of LCP, we performed transcriptomic analysis of chondrocytes treated with LCP or vehicle control (dimethyl sulfoxide, DMSO). The transcriptomic (RNA-seq) analysis revealed substantial differences in gene expression between the two groups, with clear sample clustering in the heatmap (Figure 3A). Kyoto Encyclopedia of Genes and Genomes (KEGG) pathway enrichment analysis showed that differentially expressed genes were significantly enriched in several pathways, among which the MAPK/JNK signaling pathway emerged as a major enriched category (Figure 3B). The MAPK/JNK signaling pathway is known to play a pivotal role in regulating chondrocyte senescence and mitigating OA progression [9]. Based on these findings, we next investigated whether LCP directly modulates this pathway.

WB analysis demonstrated that LCP markedly reduced the expression of phosphorylated MAPK/JNK (p-MAPK/JNK) in both H_2_O_2_-induced and naturally senescent chondrocytes, suggesting effective inhibition of MAPK/JNK activation (Figure 3C, **Figure S3A**). Isoform-specific analysis further revealed that LCP selectively inhibited the activation of MAPK8/JNK1 and MAPK9/JNK2, without affecting MAPK10/JNK3 **(Figure S3B-C)**. To confirm the functional involvement of the MAPK/JNK pathway, we employed the specific MAPK/JNK inhibitor JNK-IN-8. Pharmacological blockade of MAPK/JNK signaling abolished the anti-senescent effects of LCP, suggesting that its activity is dependent on MAPK/JNK signaling. This was evident by the unchanged expression of the senescence markers observed upon co-treatment, as shown by WB analysis (CDKN2A/p16^INK4a^, CDKN1A/p21, TRP53/p53, and γH2AX) (Figure 3D), SA-GLB1/β-gal staining (Figure 3E,F), and qRT-PCR analysis of senescence-related genes (*Gadd45 and Il6*) (Figure 3G,H). Immunofluorescence staining further confirmed that LCP significantly reduced the expression of CDKN2A/p16^INK4a^, with no additive effect observed in the presence of JNK-IN-8 (Figure 3I,J). Together, these results suggest that LCP exerts its anti-senescent effects by suppressing the MAPK/JNK signaling pathway, as identified through transcriptomic analysis and validated by molecular and cellular assays.

### LCP inhibits the MAPK/JNK pathway and chondrocyte senescence by promoting autophagic degradation of MAP2k4/MKK4

**Figure 4.**
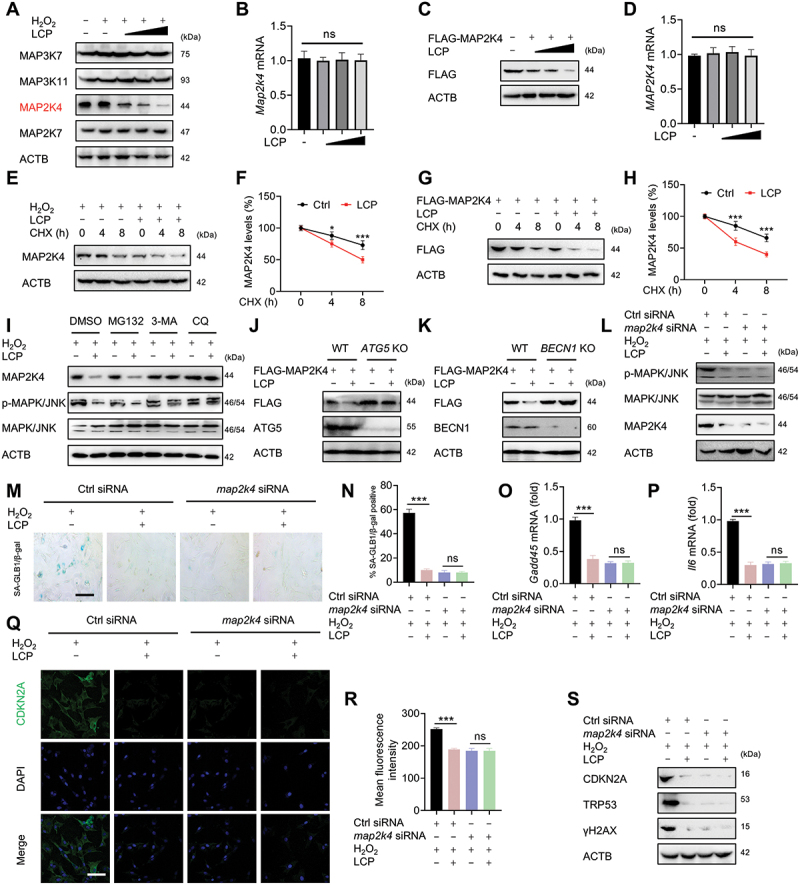
*LCP promotes MAP2K4/MKK4 degradation via the autophagy-lysosome pathway to inhibit MAPK/JNK signaling and chondrocyte senescence*. (**A**) WB analysis of MAP3K7/TAK1, MAP3K11/MLK3, MAP2K4/MKK4, and MAP2K7/MKK7 in H_2_O_2_-induced chondrocytes treated with LCP, revealing that only MAP2K4/MKK4 protein levels were significantly downregulated. (**B**) qRT-PCR analysis showing no significant change in *Map2k4/Mkk4* mRNA levels following LCP treatment in chondrocytes. (**C**) WB analysis of exogenously expressed MAP2K4/MKK4 in HEK293T cells treated with LCP. (**D**) qRT-PCR analysis of *MAP2K4/MKK4* mRNA in HEK293T cells following LCP treatment. (**E-F**) Cycloheximide (CHX) chase assay showing accelerated degradation of endogenous MAP2K4/MKK4 in chondrocytes by LCP, with corresponding quantification. (**G-H**) CHX chase assay demonstrating enhanced degradation of FLAG-MAP2K4/MKK4 in HEK293T cells by LCP, with quantification. (**I**) Effects of MG132 (proteasome inhibitor, 10 μM), 3-MA (autophagy inhibitor, 10 mM), and chloroquine (CQ, lysosomal inhibitor, 50 μM) on LCP-induced MAP2K4/MKK4 degradation in chondrocytes were evaluated. (**J-K**) WB analysis of MAP2K4/MKK4 degradation in autophagy-deficient HEK293T cells (*ATG5* or *BECN1/Beclin 1 knockout*) treated with LCP. (**L**) WB analysis of MAPK/JNK phosphorylation in H_2_O_2_-induced senescent chondrocytes with or without *map2k4/mkk4* knockdown and LCP treatment. (**M-N**) SA-GLB1/β-gal staining and quantification in H_2_O_2_-induced chondrocyte senescence with or without *map2k4/mkk4* silencing and LCP treatment. (**O-P**) qRT-PCR analysis of *Gadd45* and *Il6* expression, (**Q-R**) immunofluorescence staining and quantification of CDKN2A/p16^INK4a^, and (**S**) WB analysis of senescence markers (CDKN2A/p16^INK4a^, TRP53/p53, and γH2AX) under the same conditions. Scale bar: 100 µm. Data are expressed as mean ± SD. * *p* < 0.05, *** *p* < 0.001. ns = not significant.

To further elucidate how LCP modulates MAPK/JNK signaling, we systematically examined its effects on upstream kinases within the pathway. In chondrocytes, H_2_O_2_ stimulation robustly activated the MAP3K7/TAK1-MAP3K11/MLK3-MAP2K4/MKK4-MAP2K7/MKK7-MAPK/JNK signaling cascade, as indicated by increased phosphorylation of each component (**Figure S4A**). Among these, LCP selectively reduced H_2_O_2_-induced MAP2K4/MKK4 protein levels in a dose-dependent manner, while MAP3K7/TAK1, MAP3K11/MLK3, and MAP2K7/MKK7 protein levels remained unchanged (Figure 4A). Notably, qRT-PCR analysis revealed no significant alterations in *Map2k4/Mkk4* mRNA levels after LCP treatment (Figure 4B), suggesting that the regulation occurs at the post-transcriptional level. This post-transcriptional effect was further confirmed in HEK293T cells transfected with exogenous MAP2K4/MKK4, where LCP similarly decreased MAP2K4/MKK4 protein levels without affecting MAP3K7/TAK1, MAP3K11/MLK3, or MAP2K7/MKK7 expression (Figure 4C, **Figure S4B-D**). Consistently, *MAP2K4/MKK4* mRNA levels remained stable in HEK293T cells (Figure 4D).

To explore whether LCP promotes MAP2K4/MKK4 degradation, cycloheximide (CHX) chase assays were performed. LCP significantly enhanced the degradation rate of both endogenous MAP2K4/MKK4 in chondrocytes (Figure 4E,F) and exogenous MAP2K4/MKK4 in HEK293T cells (Figure 4G,H), confirming a direct role in promoting MAP2K4/MKK4 protein turnover. To identify the pathway responsible for this degradation, we applied MG132 (a proteasome inhibitor), 3-methyladenine (3-MA, an autophagy inhibitor), and chloroquine (CQ, a lysosomal inhibitor). While MG132 failed to reverse MAP2K4/MKK4 degradation, both 3-MA and CQ effectively reversed LCP-induced MAP2K4/MKK4 downregulation in chondrocytes (Figure 4I) and HEK293T cells **(Figure S4E)**, suggesting that LCP facilitates MAP2K4/MKK4 degradation *via* the autophagy-lysosome pathway. This was further substantiated using autophagy-deficient HEK293T cells. In cells lacking *ATG5* or *BECN1/Beclin 1*, LCP failed to promote MAP2K4/MKK4 degradation (Figure 4J,K), confirming that intact autophagic machinery is essential for this process. Functionally, siRNA-mediated knockdown of *map2k4/mkk4* resulted in only partial suppression of H_2_O_2_-induced MAPK/JNK phosphorylation **(Figure S4F)**, suggesting that while MAP2K4*/*MKK4 contributes significantly to MAPK/JNK activation under oxidative stress, it is not the sole upstream kinase involved. However, as shown in Figure 4L, LCP treatment did not provide additional suppression of p-MAPK/JNK in *map2k4/mkk4*-silenced cells, indicating that MAP2K4/MKK4 is the main mediator of LCP-induced inhibition of MAPK/JNK pathway activation. Moreover, silencing *map2k4/mkk4* suppressed chondrocyte senescence, as evidenced by decreased SA-GLB1/β-gal activity (Figure 4M,N), reduced expression of *Gadd45* and *Il6* as determined by qRT-PCR (Figure 4O,P), lowered CDKN2A/p16^INK4a^ expression confirmed by immunofluorescence (Figure 4Q,R), and downregulation of senescence-associated proteins of CDKN2A/p16^INK4a^, CDKN1A/p21, TRP53/p53, and γH2AX as shown by WB (Figure 4S). Notably, LCP treatment did not exert additional anti-senescent effects in *map2k4/mkk4*-silenced cells, further supporting that MAP2K4/MKK4 is a key upstream target of LCP in suppressing chondrocyte senescence through inhibition of the MAPK/JNK pathway. In conclusion, LCP promotes the autophagy-dependent degradation of MAP2K4/MKK4, thereby inhibiting MAPK/JNK signaling and preventing chondrocyte senescence.

### LCP mediates the autophagic degradation of MAP2K4/MKK4 via cargo receptor CCDC50

**Figure 5.**
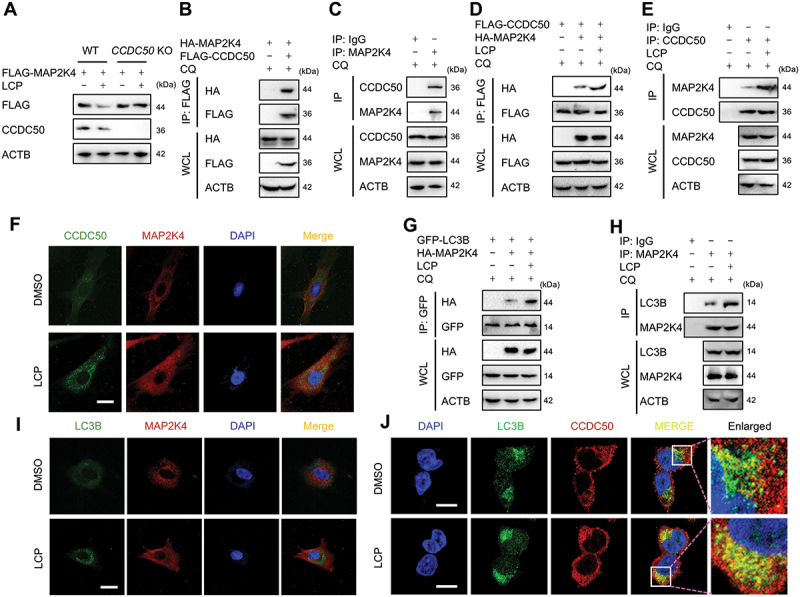
*LCP mediates autophagic degradation of MAP2K4/MKK4 via the cargo receptor CCDC50*. (**A**) WB analysis showing that LCP-induced MAP2K4/MKK4 degradation was abolished in *CDCC50* knockout (KO) HEK293T cells. (**B-C**) Co-IP assays demonstrated a direct interaction between CCDC50 and MAP2K4/MKK4 in (**B**) HEK293T cells and (**C**) chondrocytes. (**D-E**) Co-IP assays demonstrated that LCP enhances the interaction between CCDC50 and MAP2K4/MKK4 in both (**D**) overexpression exogenous and (**E**) endogenous systems. (**F**) Immunofluorescence staining revealed increased colocalization of CCDC50 and MAP2K4/MKK4 in chondrocytes after LCP treatment. (**G-H**) Co-IP analysis showing enhanced interaction between MAP2K4/MKK4 and the autophagy marker LC3B in (**G**) HEK293T cells and (**H**) chondrocytes following LCP treatment. (**I**) Immunofluorescence confirmed increased MAP2K4/MKK4-LC3B colocalization in chondrocytes treated with LCP. (**J**) Confocal microscopy showed that LCP enhanced colocalization between CCDC50 and LC3. Scale bar: 10 μm.

Cargo receptors play a critical role in selective autophagy by recognizing and delivering specific substrates into autophagosomes [11]. To identify the cargo receptor mediating LCP-induced MAP2K4/MKK4 degradation, the genes encoding several well-characterized autophagy receptors, including *SQSTM1/p62, CALCOCO2/NDP52, NBR1, TOLLIP, and CCDC50*, were individually knocked out. Among these, only *CCDC50* knockout effectively reversed LCP-induced MAP2K4/MKK4 degradation (Figure 5A), whereas knockout of the other receptor genes had no appreciable effect (**Figure S5A-D**). Co-immunoprecipitation (co-IP) assays confirmed that MAP2K4/MKK4 interacts directly with CCDC50 in both exogenous systems (HEK293T cells) (Figure 5B) and endogenous chondrocytes (Figure 5C, **Figure S5E**). Notably, LCP treatment markedly enhanced this interaction in both exogenous (Figure 5D) and endogenous systems (Figure 5E). These findings were corroborated by immunofluorescence analysis, which revealed increased colocalization of MAP2K4/MKK4 and CCDC50 in chondrocytes upon LCP treatment (Figure 5F). Moreover, LCP promoted the interaction between MAP2K4/MKK4 and the autophagy marker LC3B, a core autophagy marker, as demonstrated by co-IP in both exogenous and endogenous settings (Figure 5G,H) and further supported by fluorescence colocalization analysis (Figure 5I). In addition, fluorescence colocalization analysis revealed enhanced colocalization of CCDC50 and LC3 in chondrocytes following LCP treatment (Figure 5J). Taken together, these results demonstrate that CCDC50 acts as a selective autophagy cargo receptor responsible for mediating the degradation of MAP2K4/MKK4, and this interaction is significantly potentiated by LCP treatment.

### LCP enhances CCDC50 palmitoylation at Cys18

**Figure 6.**
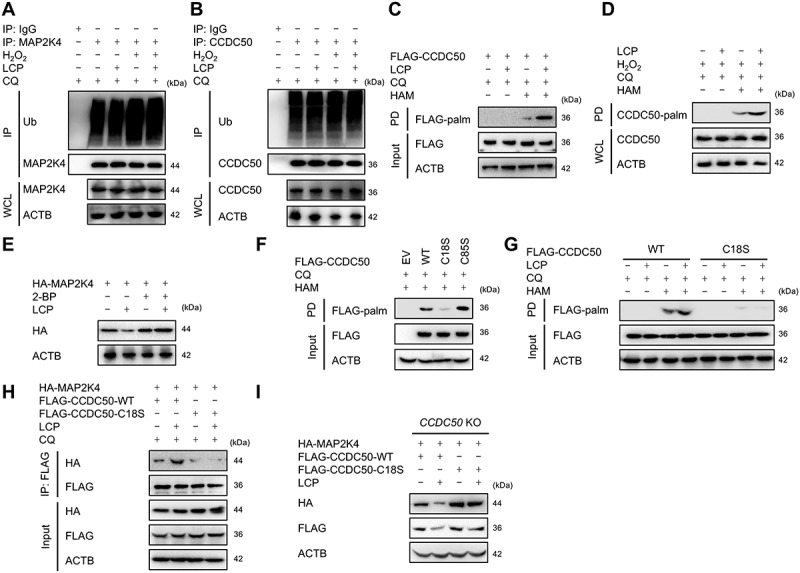
*LCP enhances CCDC50 palmitoylation at Cys18 to promote MAP2K4/MKK4 degradation*. (**A-B**) Co-IP assays indicated that LCP treatment did not alter ubiquitination (ub) levels of (**A**) MAP2K4/MKK4 or (**B**) CCDC50 in chondrocytes. (**C-D**) Acyl-biotin exchange (ABE) assays showed that LCP treatment significantly increased palmitoylation (palm) of CCDC50 in (**C**) HEK293T cells and (**D**) chondrocytes. (**E**) WB analysis showed that the palmitoylation inhibitor 2-bromopalmitate (2-BP) blocked LCP-induced HA-MAP2K4/MKK4 degradation. (**F**) Mutation of Cys18 (C18S) in CCDC50 abolished palmitoylation, while the Cys85 mutation had no effect. (**G**) ABE assays showed that the Cys18 mutation blocked LCP-induced palmitoylation of CCDC50. (**H**) Co-IP analysis demonstrated that the Cys18 mutation disrupted LCP-induced interaction between CCDC50 and HA-MAP2K4/MKK4. (**I**) WB analysis showed that the Cys18 mutation reversed LCP-induced MAP2K4/MKK4 degradation in *ccdc50* knockout cells.

Selective autophagy typically involves the recognition of specific substrates, often mediated by substrate ubiquitination [22]. However, co-IP assays revealed that LCP treatment did not alter the ubiquitination status of MAP2K4/MKK4 (Figure 6A) or CCDC50 (Figure 6B) in chondrocytes under basal conditions or in the presence of H_2_O_2_-induced stress. These results suggest that LCP does not exert its regulatory effects through modulation of ubiquitination. Recent research has highlighted the crucial role of protein palmitoylation in autophagy, particularly in facilitating protein-membrane interactions [14]. To assess whether LCP affects palmitoylation, we conducted acyl-biotin exchange (ABE) assays, which showed that LCP significantly enhanced the palmitoylation of exogenous CCDC50 in HEK293T cells (Figure 6C) and endogenous CCDC50 in chondrocytes (Figure 6D), while the palmitoylation level of MAP2K4/MKK4 remained unaffected (**Figure S6A**). Furthermore, inhibition of palmitoylation using 2-Bromopalmitate (2-BP) inhibited LCP-induced MAP2K4/MKK4 degradation (Figure 6E), indicating that palmitoylation is essential for this process. Sequence alignment *via* the SwissPalm database (https://swisspalm.org/proteins) identified two conserved cysteine residues on CCDC50, Cys18 and Cys85, across human, mouse, and rat species. Site-directed mutagenesis confirmed that Cys18 was essential for CCDC50 palmitoylation and LCP function: mutation of Cys18 abolished palmitoylation (Figure 6F), eliminated LCP-mediated CCDC50 palmitoylation (Figure 6G), disrupted its interaction with MAP2K4/MKK4 (Figure 6H), and prevented subsequent MAP2K4/MKK4 degradation (Figure 6I). Importantly, this effect appeared to be specific to MAP2K4/MKK4, as the Cys18 mutation did not impair the degradation of other known CCDC50 substrates, including NLRP3, RIGI, and IFIH1/MDA5 [23,24] (**Figure S6B-D**). These findings indicate that LCP enhances CCDC50 palmitoylation specifically at Cys18, which is essential for mediating the selective autophagic degradation of MAP2K4/MKK4.

### LCP targets ZDHHC4 to catalyze CCDC50 palmitoylation

**Figure 7.**
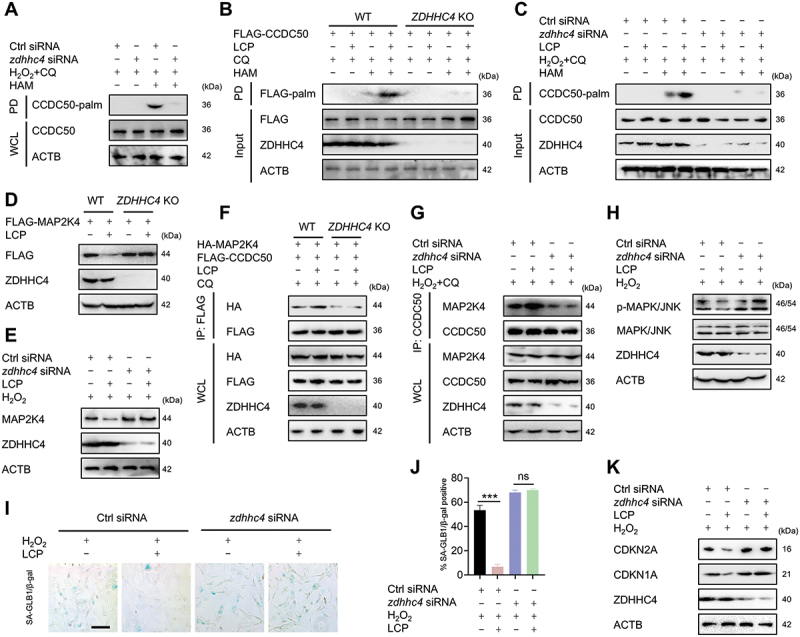
*LCP targets ZDHHC4 to catalyze CCDC50 palmitoylation*. (**A**) Knockdown of *zdhhc4* abolished palmitoylation (palm) of endogenous CCDC50 in chondrocytes. (**B**) *ZDHHC4* knockout blocked LCP-induced palmitoylation of CCDC50 in HEK293T cells. **(C)** The *zdhhc4* knockdown blocked LCP-induced palmitoylation of CCDC50 in chondrocytes. (**D**) WB analysis showed that *ZDHHC4* knockout blocked LCP-induced MAP2K4/MKK4 degradation in HEK293T cells. (**E**) WB analysis showed that *zdhhc4* knockdown prevented LCP-induced MAP2K4/MKK4 degradation in H_2_O_2_-induced chondrocytes. (**F**) Co-IP analysis revealed that the *ZDHHC4* knockout disrupted the interaction between exogenous CCDC50 and MAP2K4/MKK4. (**G**) Co-IP analysis demonstrated that knockdown of *zdhhc4* impaired the interaction between endogenous CCDC50 and MAP2K4/MKK4 in chondrocytes treated with LCP. (**H**) WB analysis showed that *zdhhc4* knockdown abolished the inhibitory effect of LCP on MAPK/JNK phosphorylation. (**I-J**) SA-GLB1/β-gal staining (**I**) and quantification (**J**) showed that *zdhhc4* knockdown reversed the anti-senescent effects of LCP. (**K**) WB analysis revealed that *zdhhc4* knockdown reversed the LCP-mediated downregulation of senescence markers CDKN2A/p16^INK4a^ and CDKN1A/p21 in chondrocytes. Scale bar: 100 μm. Data are expressed as mean ± SD. *** *p* < 0.001. ns = not significant.

Protein palmitoylation is catalyzed by the zinc finger DHHC domain-containing (ZDHHC) enzyme family in mammals, which comprises 23 members (ZDHHC1-ZDHHC24, skipping ZDHHC10) [13]. To identify the specific ZDHHC responsible for LCP-mediated CCDC50 palmitoylation, a systematic siRNA-based knockdown screen of all *ZDHHC* family members was conducted. Only *ZDHHC4* knockdown abolished CCDC50 palmitoylation **(Figure S7A-C)**. In chondrocytes, *zdhhc4* silencing markedly inhibited the palmitoylation of CCDC50 (Figure 7A). Moreover, both *ZDHHC4* knockout in HEK293T cells and siRNA-mediated knockdown in chondrocytes abolished LCP-induced CCDC50 palmitoylation (Figure 7B,C), blocked the MAP2K4/MKK4 degradation (Figure 7D,E), and disrupted the CCDC50-MAP2K4/MKK4 interaction (Figure 7F,G). Moreover, *zdhhc4* knockdown abolished the inhibitory effects of LCP on MAPK/JNK signaling in chondrocytes (Figure 7H). Functionally, the anti-senescent activity of LCP was lost upon *zdhhc4* silencing, as demonstrated by restored SA-GLB1/β-gal activity (Figure 7I,J), elevated protein expression of CDKN2A/p16^INK4a^ and CDKN1A/p21 (Figure 7K), and increased *Gadd45* and *Il6* gene expression **(Figure S7D-E)**. Collectively, these findings identify ZDHHC4 as the palmitoyltransferase targeted by LCP, which is essential for CCDC50 palmitoylation, MAP2K4/MKK4 degradation, MAPK/JNK pathway inhibition, and the suppression of chondrocyte senescence.

### LCP binds to His72 of ZDHHC4 to enhance its enzymatic activity

**Figure 8.**
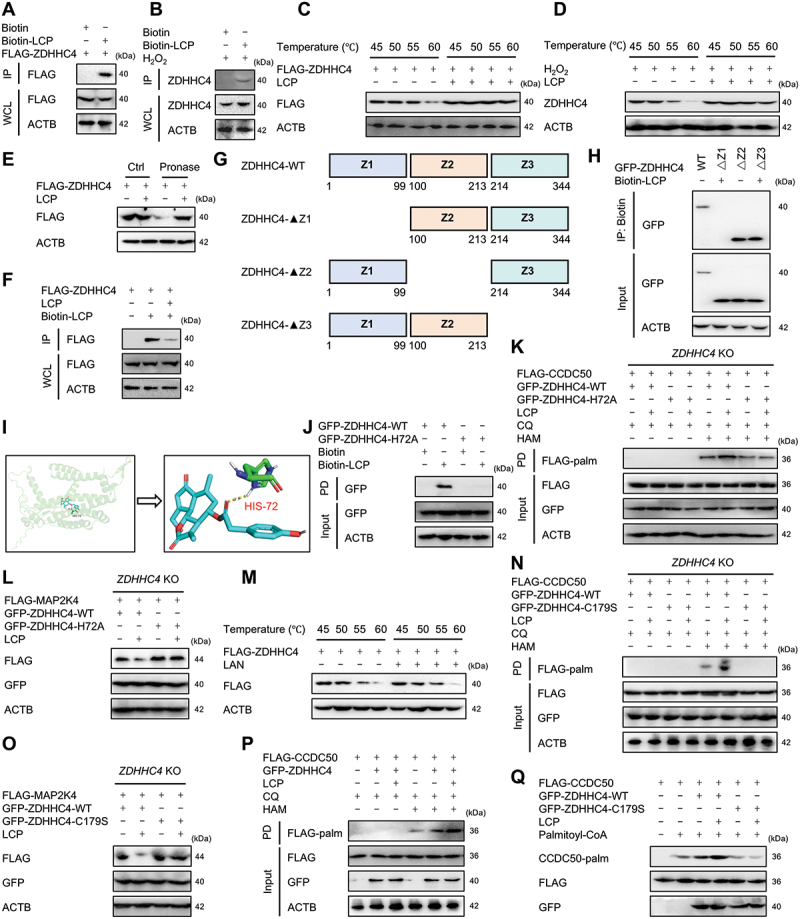
*LCP binds to His72 of ZDHHC4 to enhance enzymatic activity and promote MAP2K4/MKK4 degradation*. (**A-B**) Co-IP assays demonstrated that biotin-labeled LCP bound to ZDHHC4 through the LCP moiety itself, rather than *via* the biotin tag, in (**A**) HEK293T cells and (**B**) chondrocytes. (**C-D**) Thermal shift assays revealed that LCP treatment increased the thermal stability of both (**C**) exogenous and (**D**) endogenous ZDHHC4. (**E**) Drug affinity responsive target stability (DARTS) assays showed that LCP protected ZDHHC4 from protease digestion. (**F**) Competitive binding assays using excess unlabeled LCP showed reduced biotin-LCP binding, verifying binding specificity. (**G-H**) Domain truncation and structural analysis identified the Z1 domain as essential for LCP binding. (**I**) Molecular docking simulations predicted a key hydrogen bond interaction between LCP and the His72 residue of ZDHHC4. (**J-L**) His72 mutation (H72A) abolished biotin-LCP binding to ZDHHC4 (**J**), inhibited LCP-induced (**K**) CCDC50 palmitoylation, and (**L**) MAP2K4/MKK4 degradation. (**M**) Lactucin (LAN, a derivative lacking the p-hydroxybenzoic acid group) failed to increase ZDHHC4 thermal stability. (**N-O**) Enzymatically inactive ZDHHC4 (C179S) mutant abolished LCP-induced (**N**) CCDC50 palmitoylation and (**O**) MAP2K4/MKK4 degradation. (**P**) LCP enhanced the palmitoylation activity of ZDHHC4 on CCDC50 *in vitro*. (**Q**) *In vitro* palmitoylation assays confirmed that LCP enhanced ZDHHC4 enzymatic activity, which was abolished by the C179S mutation.

To elucidate the regulatory mechanisms underlying the effects of LCP on ZDHHC4, a series of biochemical and structural experiments was performed. Co-IP assays using biotin-labeled LCP demonstrated a direct interaction between LCP and ZDHHC4 in both HEK293T cells and chondrocytes (Figure 8A,B). This interaction was further validated by thermal shift assays and DARTS (drug affinity responsive target stability), which consistently showed that LCP binding significantly enhanced the stability of ZDHHC4, as indicated by increased melting points and reduced protease susceptibility (Figure 8C–E). Competitive binding assays using excess unlabeled LCP displaced biotin-LCP from ZDHHC4, verifying the specificity of the LCP-ZDHHC4 interaction (Figure 8F). Domain mapping experiments using truncated ZDHHC4 constructs identified the Z1 domain as the LCP-binding region (Figure 8G,H).

Molecular docking predicted a key hydrogen bond interaction between LCP and His72 within the Z1 domain (Figure 8I). Site-directed mutagenesis of His72 disrupted this binding (Figure 8J), blocked CCDC50 palmitoylation (Figure 8K), and inhibited MAP2K4/MKK4 degradation (Figure 8L), establishing the essential role of His72 in mediating LCP’s effects. Given that LCP contains a p-hydroxybenzoic acid moiety, a pharmacophore common in bioactive herbal medicines [25] and related compounds [26], we tested the relevance of this functional group. Lactucin (LAN), a structural analog of LCP lacking this moiety (**Figure S8A**), failed to stabilize ZDHHC4 (Figure 8M, **S8B**), to induce CCDC50 palmitoylation (**Figure S8C**), or to promote MAP2K4/MKK4 degradation (**Figure S8D**), indicating the critical role of the p-hydroxybenzoic acid group. To further elucidate the relationship between LCP and ZDHHC4 enzymatic activity, a catalytically inactive ZDHHC4 mutant (C179S) was employed. This mutant was incapable of mediating CCDC50 palmitoylation (**Figure S8E**) or MAP2K4/MKK4 degradation (**Figure S8F**), and LCP treatment showed no additional effect (Figure 8N,O). In contrast, LCP significantly enhanced the enzymatic activity of ZDHHC4, as shown by increased CCDC50 palmitoylation (Figure 8P). *In vitro* palmitoylation assays further validated that LCP enhanced ZDHHC4-dependent CCDC50 palmitoylation, an effect that was completely abolished by the C179S enzyme mutation (Figure 8Q). Together, these findings demonstrate that LCP directly binds to His72 of ZDHHC4 *via* its p-hydroxybenzoic acid structure, enhancing its enzymatic activity, thereby facilitating CCDC50 palmitoylation and subsequent MAP2K4/MKK4 degradation.

## Discussion

This study demonstrates that LCP mitigates OA progression through a novel mechanism involving the autophagic degradation of MAP2K4/MKK4. This process is facilitated by ZDHHC4-enhanced palmitoylation of the autophagy receptor CCDC50, which subsequently promotes selective autophagy. The degradation of MAP2K4/MKK4 through autophagy effectively suppresses MAPK/JNK signaling, thereby inhibiting chondrocyte senescence, reducing inflammation, and preventing matrix degradation associated with OA progression **(Figure S8G)**. These findings identify the ZDHHC4-CCDC50-MAP2K4/MKK4-MAPK/JNK axis as a critical target for inhibiting chondrocyte senescence and treating OA. Moreover, LCP emerges as a promising therapeutic agent that modulates autophagy to specifically counteract cellular senescence. This study also provides valuable insights into the complex interactions between autophagy, protein palmitoylation, and MAPK/JNK signaling, enhancing our understanding of their roles in the context of chondrocyte senescence and OA pathogenesis.

### LCP as a novel senomorphic drug targeting chondrocyte senescence in OA

OA is a degenerative joint disease lacking effective disease-modifying therapies, with existing treatments focusing primarily on symptomatic relief rather than addressing the underlying mechanisms of disease progression [6,27,28]. Chondrocyte senescence is increasingly recognized as a critical factor in OA pathogenesis and progression, making it a promising therapeutic target [5,6]. Senescent cells are characterized by irreversible cell cycle arrest, mitochondrial dysfunction, altered paracrine signaling, and the secretion of pro-inflammatory factors collectively referred to as the SASP [5,6,29]. Accumulation of senescent cells within joint tissues contributes to chronic inflammation, matrix degradation, and accelerated cartilage destruction. This study confirms that OA tissue samples and senescent chondrocytes exhibit hallmarks of cellular senescence, including increased SA-GLB1/β-gal activity, expression of *Cdkn2a/p16, Cdkn1a/p21, and Trp53/p53*, and SASP factors such as *Il6* and *Mmp13* [5,6,29]. Recent advances in anti-senescence therapies have provided new opportunities for OA treatment by targeting senescent cells and modulating their harmful secretory profiles [6,29]. LCP, as demonstrated in the present study, significantly reduces senescence markers and SASP factors in chondrocytes, thereby alleviating the detrimental effects of senescence on the cartilage microenvironment. These findings highlight the potential of LCP as a novel senomorphic drug for OA treatment.

### Autophagy-mediated modulation of MAPK/JNK signaling via MAP2K4/MKK4 degradation

Autophagy plays a critical role in the pathogenesis of OA, and its targeted regulation has emerged as a promising therapeutic strategy [1,2]. Reduced autophagic activity is frequently observed in OA cartilage and senescent chondrocytes, suggesting that adequate autophagic flux is essential for protecting chondrocytes and maintaining cellular homeostasis [1,2]. Enhancing autophagic activity or restoring autophagic flux with safe and effective agents may slow OA progression, reverse cellular senescence, and restore regenerative function [1–3]. The MAPK/JNK signaling pathway, activated by upstream regulators MAP2K4/MKK4 and MAP2K7/MKK7, is a key mediator of inflammation, cellular senescence, and tissue degradation under pathological conditions [7,30]. Although MAP2K7/MKK7-MAPK/JNK signaling has been studied for its role in promoting chondrocyte senescence and cartilage degradation [9], the specific role of MAP2K4/MKK4 in this cascade has been underexplored. Additionally, MAPK/JNK has been identified as a negative regulator of autophagy, further linking its dysregulation to OA progression [31]. Therefore, targeting the intersection of MAPK/JNK signaling with autophagy and senescence presents a viable strategy for alleviating OA pathology. Our findings reveal that MAP2K4/MKK4 acts as another critical regulatory node in the MAPK/JNK signaling cascade, controlling chondrocyte senescence and cartilage degeneration. LCP selectively promotes the autophagic degradation of MAP2K4/MKK4, resulting in MAPK/JNK inhibition and consequently reducing cellular senescence, inflammation, and matrix degradation, thereby effectively mitigating OA progression. This study uncovers a novel mechanism in which autophagy-mediated MAP2K4/MKK4 degradation modulates MAPK/JNK signaling to inhibit chondrocyte senescence in OA. Notably, targeting MAP2K4/MKK4 through selective autophagy with LCP provides a more specific therapeutic approach compared to broad-spectrum MAPK/JNK inhibitors, which often exhibit off-target effects.

### Palmitoylation-mediated autophagy regulation

Protein palmitoylation is a highly conserved post-translational modification that regulates protein stability, localization, trafficking, and interactions [13,14]. Although well-studied in cancer and neurodegenerative disorders, its role in chondrocyte senescence and OA remains largely underexplored. Previous studies have shown that ZDHHC5-mediated palmitoylation of NOD2 can reduce macrophage activity and alleviate OA [32]. However, the role of ZDHHC family enzymes, including ZDHHC4, in regulating chondrocyte senescence or autophagy has not been clarified. Palmitoylation may exert dual effects on autophagy regulation. On one hand, palmitoylation of substrate proteins, such as NOD2 [33], can suppress autophagic degradation by reducing their association with autophagy receptors. This effect may arise from reduced ubiquitination of substrate proteins due to altered membrane localization or impaired lysine residue accessibility [13]. Conversely, palmitoylation of key autophagy-related proteins, such as BECN1/Beclin 1 [34] or SQSTM1/p62 [35], can enhance their recognition and degradation *via* the autophagic machinery. In this study, we identified a novel mechanism by which LCP regulates autophagy through palmitoylation-dependent modulation of cargo receptor CCDC50. LCP binds to the His72 residue of ZDHHC4 *via* its p-hydroxybenzoic acid moiety, enhancing ZDHHC4 catalytic activity. This interaction promotes the palmitoylation of CCDC50 at Cys18, facilitating the selective autophagic degradation of MAP2K4/MKK4. Consequently, MAP2K4/MKK4 degradation suppresses MAPK/JNK signaling, inhibits chondrocyte senescence, and mitigates OA progression.

### CCDC50 as a novel selective autophagy receptor in OA

CCDC50 has recently been identified as a selective autophagy receptor [23]. It is known to participate in growth factor signaling, inflammation, cell death, cell proliferation, and tumorigenesis [12]. However, its role in bone and joint diseases, particularly in autophagy and senescence regulation, remains largely unexplored. This study demonstrates, for the first time, that CCDC50 acts as a selective autophagy receptor involved in chondrocyte senescence and OA. Palmitoylation of CCDC50 enhances autophagy capacity, promoting MAP2K4/MKK4 degradation, thereby inhibiting MAPK/JNK activation and chondrocyte senescence. Furthermore, this study identifies CCDC50 as a palmitoylation-regulated autophagy cargo receptor, providing new insights into the complex relationship between protein modification, autophagy, and cellular senescence. By targeting the palmitoylation-CCDC50 axis, LCP effectively regulates autophagy and suppresses senescence, highlighting its therapeutic potential in OA. Overall, this study provides new mechanistic insights and new avenues for developing targeted therapies that modulate palmitoylation and autophagy in OA treatment.

### Conclusion and future directions

This study demonstrates that LCP alleviates OA progression through a novel mechanism involving the ZDHHC4-CCDC50-MAP2K4/MKK4-MAPK/JNK axis, promoting selective autophagy and inhibiting MAPK/JNK-mediated chondrocyte senescence. By enhancing ZDHHC4-mediated palmitoylation of CCDC50, LCP facilitates the autophagic degradation of MAP2K4/MKK4, thereby suppressing MAPK/JNK signaling and mitigating senescence-related cartilage damage. These findings highlight the critical role of palmitoylation-dependent autophagy regulation, establish CCDC50 as a novel selective autophagy receptor, and identify MAP2K4/MKK4 as a key regulatory node in chondrocyte senescence and OA. The mechanistic insights presented here suggest novel therapeutic strategies for OA by targeting the ZDHHC4-CCDC50-MAP2K4/MKK4-MAPK/JNK pathway. Moreover, identification of CCDC50 as a palmitoylation-modulated autophagy receptor expands the understanding of selective autophagy regulation and provides a potential target for therapeutic intervention. Beyond elucidating the mechanism of LCP in OA, this study also contributes to a broader understanding of the interplay among autophagy, protein palmitoylation, and cellular senescence. Future investigations should explore the therapeutic potential of LCP in other degenerative diseases characterized by autophagy dysfunction and cellular senescence. Comprehensive preclinical and clinical evaluations are also warranted to assess the safety, efficacy, and long-term benefits of LCP for OA treatment and other degenerative joint diseases.

## Materials and methods

### Reagents and antibodies

The main reagents and antibodies used in this study are listed in Tables 1 and 2, respectively.

**Table 1.** The main reagents involved in this study.

Reagents	Source	Identifier
Lactucopicrin	MedChemExpress	65,725–11-3
Lactucin	MedChemExpress	1891–29-8
JNK-IN-8	MedChemExpress	1,410,880-22–6
MG132	Sigma-Aldrich	C-2211
Chloroquine	Glpbio	1954/5/7
Cycloheximide	Sigma-Aldrich	C7698
3-methyladenine	Sigma-Aldrich	5142–23-4
DMSO	Sigma-Aldrich	D2650
Paraformaldehyde	Sigma-Aldrich	P6148
Lipofectamine 2000	ThermoFisher	11,668,019
Protein G beads	GenScript	L00209
Hematoxylin and eosin dyes	Beyotime	C0105S
Masson’s trichrome kit	Solarbio	G1340
TRIZOL	Beyotime Biotechnology	R0016
Fetal bovine serum	Sigma-Aldrich	12103C
Amphotericin B	Gibco	15,290,026
Trypsinization	Gibco	R001100
Cell Counting Kit-8	Beyotime Biotechnology	C0037
Penicillin/streptomycin	Gibco	15,140,122
Lipofectamine RNAiMAX	Invitrogen	13,778,150
Enhanced chemiluminescence kit	Cell Signaling Technology	12,630

**Table 2.** The main antibodies involved in this study.

Antibodies	Source	Identifier
Anti-COL2A1	Abclonal	A1560
Anti-ADAMTS5	Abclonal	A23125
Anti-CDKN2A/p16^INK4A^	Abclonal	A0262
Anti-TRP53/p53	Cell Signaling Technology	2524S
Anti-CDKN1A/p21	Abclonal	A1483
Anti-γH2AX	Abcam	ab81299
Anti-p-MAPK/JNK	Abclonal	A4867
Anti-MAPK/JNK	Proteintech	51,153–1-AP
Anti-MAP3K7/TAK1	Proteintech	12,330–2-AP
Anti-MAP3K11/MLK3	Proteintech	11,996–1-AP
Anti-MAP3K21/MKK4	Abclonal	A7724
Anti-MAP3K20/MKK7	Abclonal	A12950
Anti-FLAG	Abclonal	AE095
Anti-BECN1	Proteintech	11,306–1-AP
Anti-ATG5	Proteintech	10,181–2-AP
Anti-CCDC50 for WB	Abclonal	A17836
Anti-CCDC50 for IP	Santa Cruz Biotechnology	sc-398994
Anti-HA	Sigma-Aldrich	05–904
Anti-GFP	Cell Signaling Technology	2956S
Anti-LC3B	Cell Signaling Technology	43,566
Anti-SQSTM1/p62	Cell Signaling Technology	88,588
anti-NBR1	Abclonal	A3949
anti-CALCOCO2/NDP52	Proteintech	12,229–1-AP
Anti-TOLLIP	Proteintech	11,315–1-AP
Anti-Biotin	Cell Signaling Technology	5597
Anti-Ub	Santa Cruz Biotechnology	sc-8017
Anti-ZDHHC4	Abclonal	A17980
Anti-ACTB/β-actin	Beijing Ray Antibody Biotech	RM2001
Anti-MAPK8/JNK1	Cell Signaling Technology	3708
Anti-MAPK9/JNK2	Cell Signaling Technology	9258
Anti-MAPK10/JNK3	Cell Signaling Technology	2305
Anti-MAPK9/JNK2 (IP)	HUABIO	ET1610-11
Anti-phosphothreonine (p-Thr) (clone H-2)	Santa Cruz Biotechnology	sc-5267
Anti-p-MAPK8/JNK1 (Thr183 and Tyr185)	Abcam	ab215208
Goat anti-rabbit IgG (H&L)	Beijing Ray Antibody Biotech	RM3002
Goat anti-mouse IgG (H&L)	Santa Cruz Biotechnology	sc-2005
Goat anti-rabbit IgG (H&L) Alexa Fluor 488	Invitrogen	A11029
Goat anti-rabbit IgG (H&L) Alexa Fluor 594	Immunoway	RS23420
Anti-Flag agarose gels	Sigma-Aldrich	A2220
Horseradish peroxidase (HRP)-anti-Flag (M2)	Sigma-Aldrich	F1804

### Establishment of mouse OA model and drug treatment

All animals were obtained from the Animal Experiment Center of Southern Medical University (Guangzhou, China) with approval from the Institutional Animal Care and Use Committee of Guangdong Second Provincial General Hospital **(No.2023-DW-KZ-005–02)**. The OA model was induced in 8–10-week-old male C57BL/6J mice using the destabilization of the medial meniscus (DMM) method [36]. Briefly, under anesthesia and sterile conditions, a medial parapatellar incision was made, and the medial meniscotibial ligament was transected with microsurgical scissors to destabilize the medial meniscus. In the sham group, the skin and joint capsule were incised, but the ligament was left intact. The incision was closed with sutures, and mice were monitored for recovery. One week post-surgery, mice received intraperitoneal injections of LCP every other day. LCP was dissolved in DMSO and appropriately diluted with sterile saline, then administered at doses of 10, 15, or 30 mg/kg, representing low-, medium-, and high-dose groups, respectively. The dosing regimen was determined with reference to the drug label and previous studies reporting the safe intraperitoneal use of LCP at doses up to 30 mg/kg in mice [37]. The control group received an equivalent volume of sterile saline as a vehicle control. The injection volume was standardized to 10 mL/kg body weight to ensure consistency across treatment groups. During the treatment period, mice were monitored for distress, joint swelling, or infection. At eight weeks post-surgery, mice were euthanized, and knee joints were collected for histological, molecular, and biochemical analyses to assess the therapeutic effects of LCP on OA progression. In addition, body weight was recorded, and blood was collected to assess potential systemic effects.

### Histological staining

Knee joints were harvested, fixed in 4% paraformaldehyde for 48 h, and decalcified in 10% ethylenediaminetetraacetic acid (EDTA) at 4°C for 4 weeks. After decalcification, the samples were embedded in paraffin and sectioned into 5-µm thick slices. Sections were stained with hematoxylin and eosin (H&E) for general morphological evaluation and with Safranin O-Fast Green (Servicebio, G1053) to assess proteoglycan content and cartilage integrity. For HE staining, sections were stained with hematoxylin for 3–5 min and then counterstained with eosin for 1–2 min, dehydrated through graded ethanol, and cleared in xylene. For Safranin O-Fast Green staining, sections were first stained with Fast Green for 5 min, differentiated in 1% acetic acid, and counterstained with Safranin O for 5–7 min. Stained sections were visualized under a light microscope, and OA severity was assessed using the OARSI scoring system [20]. Scores ranged from 0 (intact cartilage) to 6 (full-thickness erosion to subchondral bone), and statistical analyses were performed to compare the treatment groups.

### qRT-PCR analysis

The procedures followed our previous study [38,39]. Briefly, total RNA was extracted from tissues or cultured cells using the TRIzol RNA Isolation Kit (TIANGEN, DP424) following the manufacturer’s instructions. RNA concentration and purity were assessed spectrophotometrically, ensuring an A260/A280 ratio between 1.8 and 2.0. Complementary DNA (cDNA) was synthesized from 1 µg of RNA in a 20 µL reaction using a cDNA synthesis kit (NovoProtein, E047-01A). qRT-PCR was conducted on an ABI Q6 Analyzer with SYBR GreenER qRT-PCR SuperMix Universal (NovoProtein, E096-01A) and specific primers. Relative gene expression levels were determined using the ΔΔ-Ct method, normalized to the *GAPDH* housekeeping gene. Results were expressed as fold changes relative to the control group. Primer sequences are listed in **Table S1**.

### WB analysis

The experimental procedures were conducted as described in our previous studies [40,41]. Briefly, total protein was extracted from cells or tissues using an appropriate lysis buffer and quantified using the BCA^TM^ protein assay kit (Invitrogen, 23,225). Subsequently, equal amounts of protein were separated by SDS-PAGE and subsequently transferred onto a polyvinylidene difluoride (PVDF) membrane. The membrane was blocked with 10% nonfat milk prepared in TBST [Tris-buffered saline (Servicebio, G0004-1 L) with 0.1% Tween-20 (Solarbio, T8220-100)] to prevent nonspecific binding. The membrane was then incubated with the primary antibody specific to the target protein at the recommended dilution overnight at 4°C. After washing, the membrane was incubated with an HRP-conjugated secondary antibody for 1 h at room temperature. The EMD Millipore Luminata^TM^ Western HRP Chemiluminescent Substrate was used for detection according to the manufacturer’s protocol.

### Immunohistochemistry staining

The experimental procedures were performed according to our previous study [42]. Briefly, tissue sections were dewaxed, rehydrated, and underwent antigen retrieval in sodium citrate buffer (pH 6.0) for 15 min. Endogenous peroxidase was blocked with 1% H_2_O_2_ for 10 min, followed by blocking with 1% bovine serum albumin (BSA; Sigma-Aldrich, A1933-25 G) for 30 min. Sections were incubated overnight at 4°C with primary antibodies, then with secondary antibodies at room temperature for 1 h. 3,3′-diaminobenzidine (DAB) substrate (Thermo Scientific, 34,065) was applied for color development. Sections were counterstained with hematoxylin, washed, mounted, and observed under an optical microscope.

### ELISA Assay

The experiment was conducted using a 96-well ELISA plate following the manufacturer’s protocol. Samples were added to wells pre-coated with capture antibodies and incubated at 37°C for the designated time, followed by thorough washing. Enzyme-conjugated secondary antibodies were added, incubated, and washed again. Substrate solution was then introduced to initiate the color reaction, which was terminated with a stop solution after sufficient development. Absorbance was measured at 450 nm using a microplate reader, and target molecule concentrations were calculated based on a standard curve.

### Isolation and culture of chondrocytes and senescence induction

The methods followed the procedures described by Cao et al [21]. Briefly, costal and articular cartilage were isolated from newborn mice (24–72 h old) under a stereoscopic light microscope. The cartilage was digested with trypsin (Servicebio, G4001-100 ML) for 30 min, followed by digestion with 0.1% type II collagenase (Sigma-Aldrich, C6885-100 MG) containing 10% fetal bovine serum (FBS) and 100 U/mL penicillin-streptomycin at 37°C for 4–6 h. The resulting primary chondrocytes were purified, resuspended, and maintained in DMEM/F12 medium (GIBCO, C11330500BT) supplemented with 10% FBS, 100 U/mL penicillin, and 100 µg/mL streptomycin at 37°C with 5% CO_2_. For the oxidative stress-induced senescence model, primary chondrocytes were treated with 100 µM H_2_O_2_ for 1 h, followed by medium replacement and continued culture in fresh medium until 24 h post-treatment [23,24]. For the natural senescence model, primary chondrocytes were passaged until the 7^th^ generation (P7), ensuring model stability.

### Cell viability assay

Chondrocyte viability was assessed using the Cell Counting Kit-8 (CCK-8). After cells were seeded in 96-well plates and allowed to adhere, various concentrations of LCP were added and incubated for 24 h. Then, CCK-8 reagent (10 μL per well) was added and incubated for 4 h. Cell viability was determined using a microplate reader [43].

### SA-GLB1/β-gal staining

SA-GLB1/β-gal staining was conducted using the Cell Senescence β-Galactosidase Staining Kit (Solarbio, G1580, Beijing, China) following the manufacturer’s instructions. Cells were washed with PBS (Servicebio, G4202), fixed for 15 min with 70% ethanol, and incubated with the staining solution at 37°C for 16 h. SA-GLB1/β-gal-positive cells were counted in three random fields per dish, and senescence was quantified as the percentage of positive cells relative to the total cell count using ImageJ (NIH, Bethesda, MD, USA) [21].

### Immunofluorescence

Chondrocytes were cultured in 6-well plates to 70–80% confluence. The culture medium was removed, and the cells were washed twice with PBS. Cells were fixed with 4% paraformaldehyde for 10–15 min and then blocked with 1% BSA and 0.1% Triton X-100 for 1 h at room temperature. Primary antibodies were added and incubated overnight at 4°C. After washing, fluorescently labeled secondary antibodies were applied and incubated for 1 h at room temperature in the dark. Following washing again, the cells were stained with DAPI for 10 min to label the nuclei. After another wash, slides were mounted with an anti-fluorescence quencher, and images were captured using a fluorescence microscope.

### Protein inhibition experiment

Chondrocytes were seeded in 6-well plates and cultured to 70–80% confluence. Senescence was induced by treating the cells with H_2_O_2_, and LCP was added for 24 h. CHX was then added at concentrations of 10–50 µg/mL for 0–8 h to inhibit protein synthesis. Alternatively, HEK293T cells were transfected with FLAG-tagged MAP2K4/MKK4 and treated with LCP for 24 h. CHX was added for 0–8 h post-treatment to inhibit protein synthesis. After each time point, cells were collected, total protein was extracted, and MAP2K4/MKK4 protein expression was assessed with WB analysis.

### Plasmid transfection experiments

HEK293T cells (provided by Prof. Xiao Yu from Southern Medical University, China) were cultured to 70–80% confluence. The coding sequence of the target protein was cloned into the pcDNA3.1 expression vector (Sino Biological Inc, HG11175-CY) and transfected into HEK293T cells using the Lipofectamine 2000 transfection reagent. After transfection, cells were incubated for 24 h before proceeding with further experiments [43].

### Cell autophagy and protein degradation experiments

Target-aged chondrocytes or HEK293T cells were treated with 3-methyladenine (3-MA, 10 mM) or chloroquine (CQ, 50 μM) for 24 h to inhibit different stages of autophagy. To inhibit proteasomal degradation, cells were treated with MG132 (10 μM) for 24 h. After treatment, total cell protein was extracted, and protein expression was assessed by WB analysis [43].

### siRNA-mediated gene silencing experiments

Cells were seeded in 6-well plates and cultured to 70–80% confluence. Specific siRNA targeting the gene of interest was designed and diluted to a final concentration of 50 nM. The transfection system was prepared using Lipofectamine RNAiMAX according to the manufacturer’s instructions, and the siRNA-transfection complex was formed by incubating at room temperature for 10 min. The transfection mixture was added to an antibiotic-free culture medium and incubated at 37°C with 5% CO_2_ for 24 h. The sequences of all target siRNAs are provided in **Table S2.**

### Cell knockout (ko) experiment

Transfection was performed when cells reached 70–80% confluence, following the manufacturer’s instructions. After transfection, the cells were cultured at 37°C with 5% CO_2_ for 24 h to allow the Cas9 protein and sgRNA to enter the cells and initiate gene editing. Relevant experiments were then conducted. The mRNA expression level of the target gene was analyzed by qRT-PCR, and the expression of the target protein was detected by WB analysis.

### Co-IP assay

Cells were washed twice with ice-cold PBS and lysed on ice for 30 min using lysis buffer. The lysates were centrifuged, and the supernatants were collected. Primary antibodies were added to the supernatants, and the mixtures were rotated overnight at 4°C. Then, Protein A/G magnetic beads were added, and the sample was incubated and rotated for 1 h at 4°C. The immune complexes were washed 3–5 times to remove nonspecific binding proteins. The washed beads were resuspended in SDS sample buffer and heated at 100°C for 10 min to elute the bound proteins. The samples were analyzed by SDS-PAGE followed by WB to detect the target proteins and their interacting partners.

### Acyl-biotin exchange (ABE) assays

The experimental procedures were conducted as described in a previous study [44]. HEK293T cells or chondrocytes were harvested and incubated with lysis buffer containing 150 mM NaCl, 50 mM Tris-HCl (pH 7.4), 5 mM EDTA, 10 mM N-ethylmaleimide (NEM; Selleck, S3692), 2× protease inhibitors (Beyotime, P1005), 2 mM PMSF (Beyotime, ST506), and 2× phosphatase inhibitors (Beyotime, P1045) at 4°C for 2 h. After incubation, the lysates were centrifuged at 16,000 × g for 10 min, and the supernatants were collected. Proteins were precipitated using a methanol-chloroform (MC) extraction protocol (4× volume methanol, 1.5× volume chloroform, 3× volume water), followed by centrifugation at 14,000 × g for 10 min. The interphase was collected, and an additional 3× volume of methanol was added to the collected interphase and spun down at 16,000 × g for 10 min. After a final round of MC precipitation, the pellet was air-dried and dissolved in 2% SDS at room temperature for 10 min. The samples were diluted fivefold in lysis buffer containing 0.7 M hydroxylamine (HAM; Sigma-Aldrich, 431,362) and incubated for 1 h at room temperature. Following HAM incubation, the sample underwent three additional MC precipitations to remove excess HAM and was then dissolved in 2% SDS at room temperature for 10 min. The samples were incubated in lysis buffer containing 1 mM EZ-Link HPDP-biotin (Thermo Scientific, 21,341) for 1 h at room temperature to label the proteins with biotin. To remove the unreacted biotinylation reagent, an additional round of MC precipitation was performed, followed by solubilization in 2% SDS lysis buffer. The final sample was adjusted to less than 0.5% SDS and then incubated with streptavidin-agarose beads (Thermo Fisher Scientific, 20,349) overnight at 4°C. After washing four times with lysis buffer, biotinylated proteins were eluted with lysis buffer containing 2% SDS and 1% β-mercaptoethanol and incubated at room temperature for 15 min. The proteins were collected by centrifugation, further purified using chloroform/methanol precipitation, and analyzed by WB to evaluate the palmitoylation status of proteins.

### Molecular docking simulation

The experimental procedures were performed according to our previous study [11]. The SDF format file of the LCP molecular structure was retrieved from the PubChem database, and the ZDHHC4 protein structure was obtained from the Protein Data Bank (PDB) database. The target protein structure was preprocessed by removing water molecules and small ligands using PyMOL 2.1.0 software. Subsequent hydrogenation and charge assignments were carried out using AutoDock Tools 1.5.6, and the prepared structure was saved in the.pdbqt format. The ZDHHC4 protein was set as the receptor, and LCP served as the ligand. Molecular docking was performed using Vina 2.0 implemented in PyRx software to calculate the binding energy. The resulting docking poses were visualized and analyzed with PyMOL software. Binding affinity (kcal/mol) values were used to assess the strength of interaction. Generally, binding energies below −4.25 kcal/mol suggest potential binding activity, values below −5.0 kcal/mol indicate good binding activity, and values below −7.0 kcal/mol imply strong binding activity between the receptor and the ligand.

### Thermal stability experiments

HEK293T cells transfected with target proteins or H_2_O_2_-induced aged chondrocytes were treated with LCP, and then protein lysates were obtained. The lysates were subjected to heat treatments at temperatures of 45°C, 50°C, 55°C, and 60°C, with each temperature maintained for 10 min. The effect of LCP on protein thermal stability was analyzed with WB analysis.

### Drug affinity responsive target stability (DARTS) experiments

HEK293T cells transfected with the target protein ZDHHC4 were treated with LCP or lactucin. Cell lysates were prepared, and Pronase solution (0.1 mg/mL; MCE, HY-114158) was added to the lysates and incubated at 37°C for 10 min to induce protein degradation. The reaction was terminated by adding protease inhibitors. Protein degradation levels were analyzed with WB analysis.

### Statistical analysis

Data were analyzed using SPSS Statistics 20 statistical software (SPSS, Inc., Chicago, IL, USA) and GraphPad Prism version 9.0 and presented as mean ± standard deviation (SD). Sample size estimation was conducted using PASS software (version 15, NCSS, LLC, Kaysville, UT, USA), based on a one-way ANOVA design. The parameters were set as follows: significance level (α) = 0.05, statistical power (1 - β) = 0.80, and effect size (Cohen’s f = 0.7). This calculation indicated that 6 animals per group were sufficient to detect statistically significant group differences in the primary outcome, which was histological evaluation of the knee joints. To accommodate additional downstream analyses, 16 mice were ultimately included in each group. Specifically, 6 animals were used for histological evaluation, 5 for RNA extraction and qRT-PCR analysis, and 5 for protein extraction and WB analysis. Data were assessed for normality using the Shapiro-Wilk test and for homogeneity of variances using Levene’s test. If both assumptions were met, comparisons between the two groups were performed using two-tailed Student’s t-tests. For multiple group comparisons, one-way ANOVA followed by Tukey’s post-hoc tests was applied. If data failed to meet assumptions of normality or equal variance, non-parametric tests were used: the Mann-Whitney U test for two-group comparisons and the Kruskal-Wallis test followed by Dunn’s post-hoc test for multiple group comparisons. A *p*-value < 0.05 was considered statistically significant.

## Supplementary Material

Supplementary Materials R5.docx

## Data and materials availability

All data are available in the main text or the supplementary materials.
