# Supplementary material for: Lactucopicrin promotes the autophagic degradation of MAP2K4/MKK4 by mediating CCDC50 palmitoylation to alleviate osteoarthritis progression
Source: Autophagy. 2026 Jan 21;22(3):526–44. doi: 10.1080/15548627.2025.2601041 (PMC12931897; doi:10.1080/15548627.2025.2601041)
Supplement: Supplementary Materials R5.docx [file KAUP_A_2601041_SM1665.docx]

**Lactucopicrin Promotes the Autophagic Degradation of MAP2K4/MKK4 by Mediating CCDC50 Palmitoylation to Alleviate Osteoarthritis Progression**

**This file includes:**

**1. Supplementary Methods**

**1.1 Pharmacokinetic Analysis**

**1.2 Human Chondrocyte Isolation**

**2. Supplementary Table**

**Table S1**: Primers for qRT-PCR used in this study.

**Table S2** shows the sequence of all target siRNAs.

**3. Supplementary Figures**

**Figure S1.** Systemic safety profile and pharmacokinetics of lactucopicrin (LCP) *in vivo*.

**Figure S2.** LCP inhibits chondrocyte senescence *in vitro*.

**Figure S3.** LCP inhibits chondrocyte senescence *via* the MAPK/JNK signaling pathway.

**Figure S4.** LCP promotes MAP2K4/MKK4 degradation *via* the autophagy-lysosome pathway to inhibit MAPK/JNK signaling and chondrocyte senescence.

**Figure S5.** LCP mediates autophagic degradation of MAP2K4/MKK4 *via* the cargo receptor CCDC50.

**Figure S6.** LCP enhances CCDC50 palmitoylation at Cys18 to promote MAP2K4/MKK4 degradation.

**Figure S7.** LCP inhibits chondrocyte senescence by targeting ZDHHC4 to catalyze the CCDC50 palmitoylation.

**Figure S8.** LCP binds to His72 of ZDHHC4 to enhance enzymatic activity and promote MAP2K4/MKK4 degradation.

**1. Supplementary Methods**

***1.1 Pharmacokinetic analysis.***

***Animal Model and Sample Collection.***

Male C57BL/6 mice (8 weeks old) were used to establish an osteoarthritis (OA) model *via* destabilization of the medial meniscus surgery on the right knee. Four weeks post-surgery, a single intraperitoneal injection of Lactucopicrin (LCP, 15 mg/kg) was administered. Mice were euthanized at 0, 2, 6, and 24 h after administration (n ≥ 3 per time point). Knee joint cartilage was harvested, immediately snap-frozen in liquid nitrogen, and stored at -80°C until further analysis.

***Tissue Sample Preparation.***

Approximately 100 mg of knee cartilage tissue was weighed and homogenized in 400 μL methanol with three stainless steel beads (QIAGEN, 69989) using a tissue homogenizer (Jingxin, JX-GN150; 25 Hz, 2 min per cycle, 2 cycles). The homogenates were centrifuged at 1,000 x g for 10 min at 4°C. Supernatants (200 μL) were collected and lyophilized at -80°C to complete dryness. Dried residues were reconstituted in 100 μL of 50% methanol: water, vortexed, and centrifuged at 1,000 x g for 10 min. The final supernatants were subjected to LC-MS/MS analysis.

***Preparation of calibration standards and quality control samples.***

LCP stock solution was prepared in 50% methanol: water and serially diluted to obtain calibration standards at concentrations of 1, 5, 10, 20, 50, 200, 500, and 1000 ng/mL. Each concentration level was prepared in triplicate. Quality control (QC) samples at low, medium, and high concentrations were also prepared to assess the accuracy and precision of the analytical method. All solutions were stored at -20°C until use.

***Chromatographic analysis.***

Liquid chromatographic separation was carried out using an UltiMate 3000 UHPLC system (Thermo Scientific) equipped with an ACQUITY UPLC BEH C18 column (1.7 μm, 2.1 × 100 mm; Waters, 186009461). The mobile phase consisted of 0.1% formic acid in water (solvent A) and 0.1% formic acid in acetonitrile (solvent B), delivered at a constant flow rate of 0.35 mL/min. The column temperature was maintained at 40°C, and the injection volume was 5 μL. The gradient elution program was as follows: 0-3 min, B increased linearly from 10% to 90%; 3-5 min, held at 90% B; 5.1 min, returned to 10% B and re-equilibrated until 7 min.

***Mass spectrometry analysis.***

Mass spectrometric analysis was performed using a Q-Exactive high-resolution mass spectrometer (Thermo Fisher Scientific) equipped with a heated electrospray ionization (HESI) source, operating in positive ion mode. The ion source parameters were set as follows: spray voltage, 3.0 kV; capillary temperature, 320°C; ion transfer tube temperature, 310°C; sheath gas flow rate, 30 arbitrary units; auxiliary gas flow rate, 10 arbitrary units. Full scan mode was used with a scan range of m/z 100-800, resolution of 70,000, AGC target of 3 × 10⁶, and a maximum injection time of 200 ms.

***1.2 Human chondrocyte isolation.***

Human articular cartilage samples were collected from two patients with OA, an old female and an old male, who underwent unicompartmental knee arthroplasty at the Affiliated Guangdong Second Provincial General Hospital of Jinan University. Both patients were clinically diagnosed with unicompartmental OA involving a single compartment of the knee joint. Intact cartilage was harvested from the non-weight-bearing (undamaged) compartment. Sample collection was conducted following approval from the institutional ethics committee (Approval No. 2025-KY-KZ-247-01), and all procedures adhered to institutional and ethical guidelines. The harvested cartilage tissue was subsequently used for chondrocyte isolation and *in vitro* aging-related analyses.

**Table S1.** Primers used for qRT-PCR in this study.

| **Gene** | **Sequences (5′-3′)** | **Species** |
| --- | --- | --- |
| *Col10a1/ColX* | Forward: AAAGCTTACCCAGCAGTAGG  Reverse: ACGTACTCAGAGGAGTAGAG | Mice |
| *Mmp13* | Forward: CTTCTTCTTGTTGAGCTGGACTC  Reverse: CTGTGGAGGTCACTGTAGACT | Mice |
| *Adamts5* | Forward: GGAGCGAGGCCATTTACAAC  Reverse: CGTAGACAAGGTAGCCCACTTT | Mice |
| *Cdkn1a/p21* | Forward: GGGCGCACGATGTTCAGAA  Reverse: CACCACCAGGTCGAAATGGG | Mice |
| *Cdkn2a/p16* | Forward: GCTCAACTACGGTGCAGATTC  Reverse: GCACGATGTCTTGATGTCCC | Mice |
| *Gadd45* | Forward: GGCCGAGGAGCAAGAATGG  Reverse: CATGCACTCTGCGATACGCT | Mice |
| *Il1a* | Forward: CGAAGACTACAGTTCTGCCATT  Reverse: GACGTTTCAGAGGTTCTCAGAG | Mice |
| *Cxcl1* | Forward: CTGGGATTCACCTCAAGAACATC  Reverse: CAGGGTCAAGGCAAGCCTC | Mice |
| *Gapdh* | Forward: ATACGGCTACAGCAACAGGG  Reverse: TGTGAGGGAGATGCTCAGTG | Mice |
| *CXCL1* | Forward: AGCTTGCCTCAATCCTGCATCC  Reverse: TCCTTCAGGAACAGCCACCAGT | Human |
| *CDKN1A /p21* | Forward: AGGTGGACCTGGAGACTCTCAG  Reverse: TCCTCTTGGAGAAGATCAGCCG | Human |
| *CDKN2A/p16* | Forward: CTCGTGCTGATGCTACTGAGGA  Reverse: GGTCGGCGCAGTTGGGCTCC | Human |
| *GADD45* | Forward: CTGGAGGAAGTGCTCAGCAAAG  Reverse: AGAGCCACATCTCTGTCGTCGT | Human |
| *IL6* | Forward: AGACAGCCACTCACCTCTTCAG  Reverse: TTCTGCCAGTGCCTCTTTGCTG | Human |
| *IL1A* | Forward: TGTATGTGACTGCCCAAGATGAAG  Reverse: AGAGGAGGTTGGTCTCACTACC | Human |
| *GAPDH* | Forward: GTCTCCTCTGACTTCAACAGCG  Reverse: ACCACCCTGTTGCTGTAGCCAA | Human |

**Table S2.** Sequence of target siRNAs used in this study.

| **Gene** | **Target sequences (5′-3′)** |
| --- | --- |
| *map2k4/mkk4* siRNA | TTCTGTGAAAAGGCACAAAGTAA |
| *ZDHHC1* siRNA | ATCTACTATGTACAAGATGAACA |
| *ZDHHC2* siRNA | TGGCGTCAATGCACTTGTTAAAA |
| *ZDHHC3* siRNA | GCGAGAACAACCAGAAGTACTTC |
| *ZDHHC4* siRNA | CACAGATATTTTCCTGTATAATT |
| *ZDHHC5* siRNA | AAGAAAGATAAGAGACATTGACT |
| *ZDHHC6* siRNA | ATGGTGTAATCTGCGTTAACAGT |
| *ZDHHC7* siRNA | AGGACAGTATGCACCTTAAGATC |
| *ZDHHC8* siRNA | GTCGAAACTATCGCTACTTCTTC |
| *ZDHHC9* siRNA | ACGGATTTTGATCTTTGTTCTTC |
| *ZDHHC11* siRNA | AGCCATACTCAATAATGAAAAGC |
| *ZDHHC12* siRNA | TGCACACTTTTACAAATTTAATA |
| *ZDHHC13* siRNA | CTCTAGTAACTGTGACATTGTCA |
| *ZDHHC14* siRNA | CGGGAAGAAACAAGTTCTTCTGT |
| *ZDHHC15* siRNA | CGGGAAGAAACAAGTTCTTCTGT |
| *ZDHHC16* siRNA | CTGGAATCTGATCCTGATTGTCT |
| *ZDHHC17* siRNA | AGCCATTATAACCATGGATATGG |
| *ZDHHC18* siRNA | GCCTCTTCTTCGTCTTTGACTGT |
| *ZDHHC19* siRNA | ACCTTCTTCAGTCTTGTTTCACT |
| *ZDHHC20* siRNA | GCGTGTGAAAGACCGTTGTTTAC |
| *ZDHHC21* siRNA | GCGACGAATGAAGAACTTTTTCA |
| *ZDHHC22* siRNA | CAGGAACATGCGCAACTTCGTCC |
| *ZDHHC23* siRNA | ACCTATTTACGAGATGTAAGTTG |
| *ZDHHC24* siRNA | CGCAAACACTCCAATTTCTCTGG |


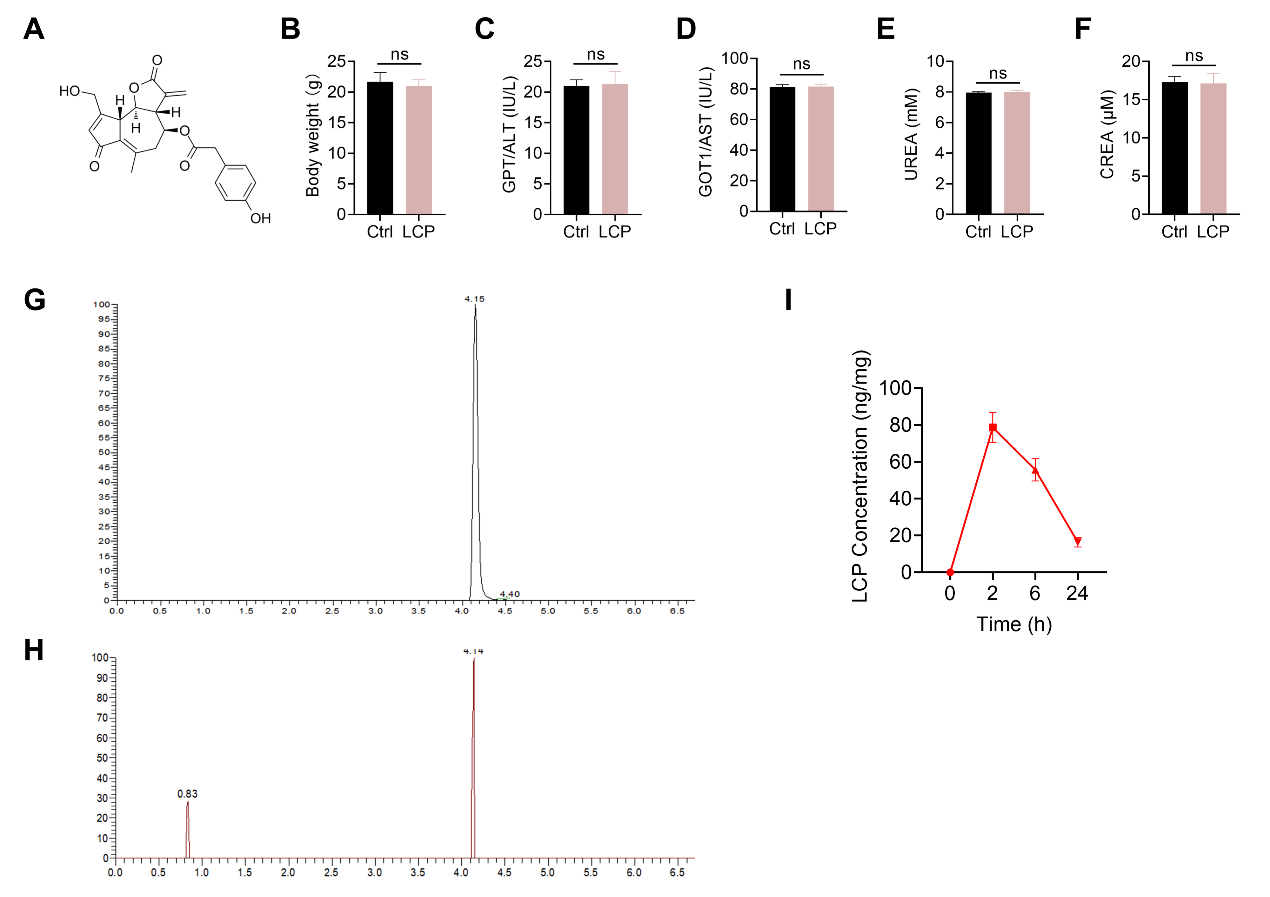


**Figure S1.** Systemic safety profile and pharmacokinetics of lactucopicrin (LCP) in vivo. (**A**) The chemical structure of LCP. (**B**) Body weight assessment of mice after high-dose LCP treatment. (**C-F**) Serum biochemical markers of hepatic and renal function after high-dose LCP treatment: GPT/ALT, GOT1/AST, urea, and creatinine (CREA). (**G-H**) Representative LC-MS/MS chromatograms showing the detection of LCP in cartilage tissue. (**I**) Pharmacokinetic profile of LCP in cartilage following intraperitoneal injection administration, showing rapid tissue distribution and peak concentration at 2 h, with retention up to 24 h. Data are presented as mean ± SD. ns = not significant.


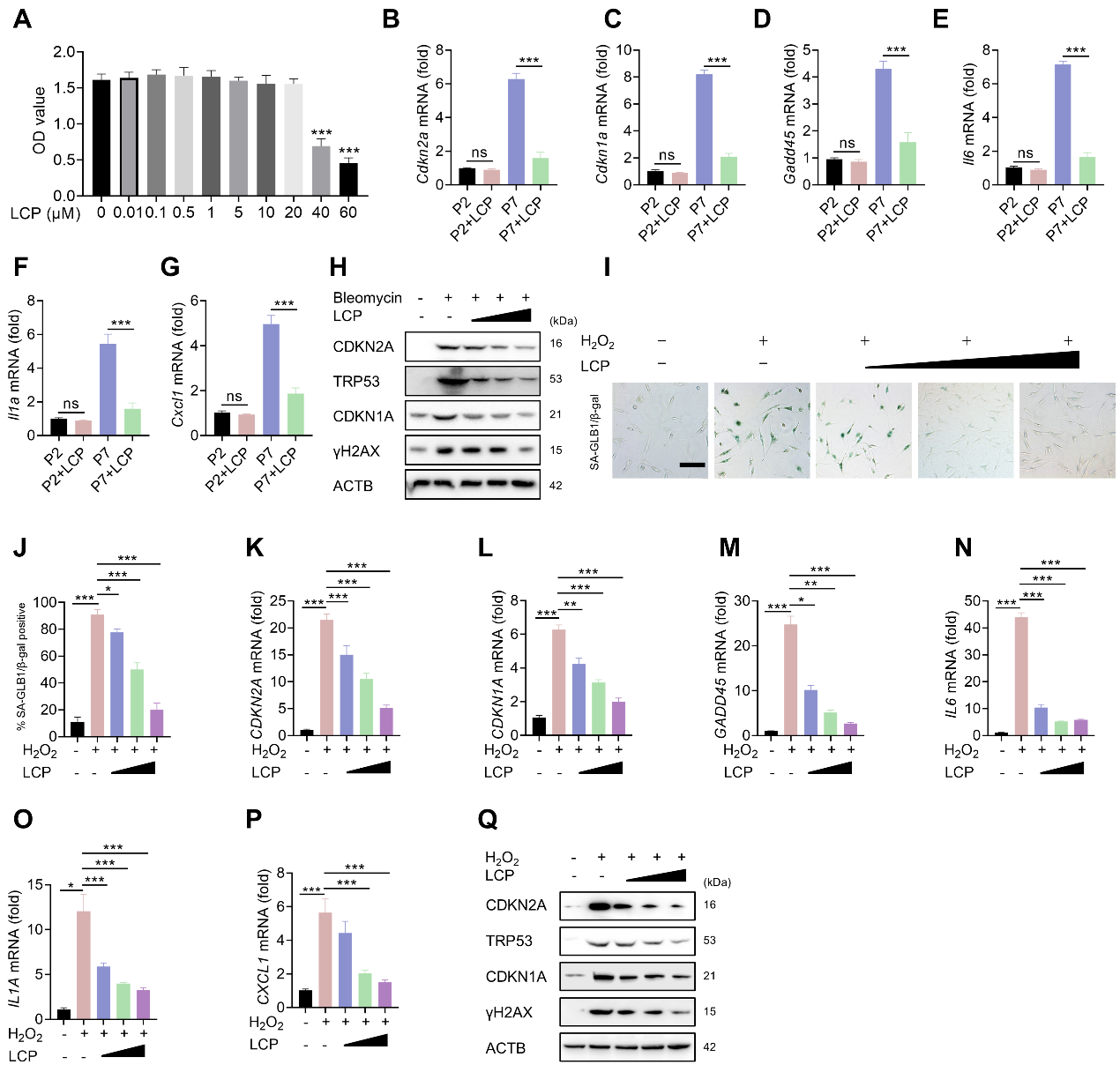


**Figure S2.** LCP inhibits chondrocyte senescence in vitro. (**A**) The CCK-8 assay was used to determine the safe therapeutic range of LCP by assessing cell viability. (**B-G**) qRT-PCR analysis was used to assess the senescence-related genes (*Cdkn2a/p16, Cdkn1a/p21, Gadd45*) and SASP factors (*Il6, Il1a, Cxcl1*) in the mouse chondrocyte senescence model (passage 7). (**H**) WB analysis was used to assess the effect of LCP on the expression levels of senescence markers (CDKN2A/p16^INK4A^, CDKN1A/p21, TRP53/p53, and γH2AX) in a bleomycin-induced chondrocyte senescence model. (**I-J**) Representative images of SA-GLB1/β-gal staining in H_2_O_2_-induced senescent human chondrocytes treated with LCP and corresponding quantification of SA-GLB1/β-gal positive cells. (**K-P**) qRT-PCR analysis of *CDKN2A/p16*, *CDKN1A/p21*, *GADD45*, *IL6, IL1A*, and *CXCL1* expression levels in H₂O₂-induced senescent human chondrocytes treated with LCP. (**Q**) WB analysis of senescence markers in H_2_O_2_-induced senescent human chondrocytes treated with LCP. Data are presented as mean ± SD. ***p < 0.001. ns = not significant. Scale bars: 100 µm.


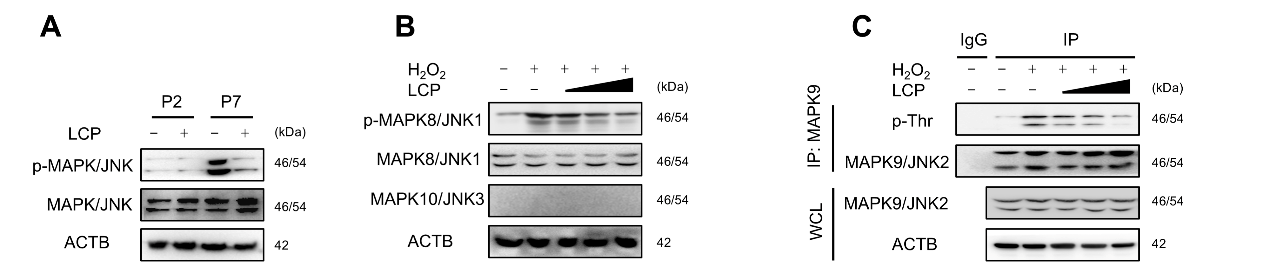


**Figure S3.** LCP inhibits chondrocyte senescence via the MAPK/JNK signaling pathway. (**A**) WB analysis of phosphorylated MAPK/JNK (p-MAPK/JNK) and total MAPK/JNK levels in a naturally senescent chondrocyte model (P7), with or without LCP treatment. (**B**) WB analysis of phosphorylated MAPK8/JNK1 (p-MAPK8/JNK1), total MAPK8/JNK1, and MAPK10/JNK3 expression in H_2_O_2_**-**induced chondrocyte senescence treated with LCP. (**C**) Co-IP analysis of phospho-threonine (p-Thr) to assess MAPK9/JNK2 activation in H_2_O_2_-induced chondrocyte senescence following LCP treatment.


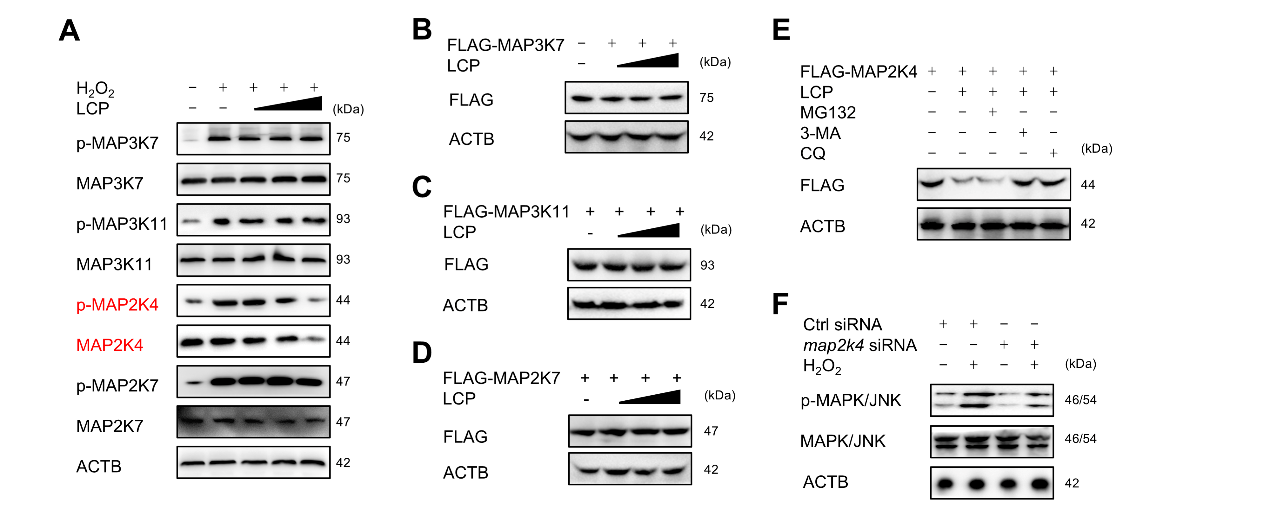


**Figure S4.** LCP promotes MAP2K4/MKK4 degradation via the autophagy-lysosome pathway to inhibit MAPK/JNK signaling and chondrocyte senescence. (**A**) WB analysis of phosphorylated and total levels of MAP3K7/TAK1, MAP3K11/MLK3, MAP2K4/MKK4, and MAP2K7/MKK7 in H_2_O_2_-induced chondrocytes treated with LCP. (**B-D**) WB analysis of HEK293T cells overexpressing FLAG-tagged MAP3K7/TAK1 (**B**), MAP3K11/MLK3 (**C**), and MAP2K7/MKK7 (**D**) treated with LCP, confirming that LCP does not significantly alter the protein levels of these kinases. (**E**) WB analysis of FLAG-MAP2K4/MKK4 in HEK293T cells treated with LCP in the presence of the proteasome inhibitor MG132 (10 μM), autophagy inhibitor 3-MA (10 mM), or lysosomal inhibitor CQ (50 μM), demonstrating that MAP2K4/MKK4 degradation is dependent on the autophagy-lysosome pathway. (**F**) WB analysis of MAPK/JNK phosphorylation in H_2_O_2_-induced chondrocytes transfected with control or *map2k4/mkk4* siRNA, showing that knockdown of *map2k4/mkk4* only partially suppresses MAPK/JNK activation, indicating MAP2K4/MKK4 is a key but non-exclusive upstream kinase in MAPK/JNK activation.


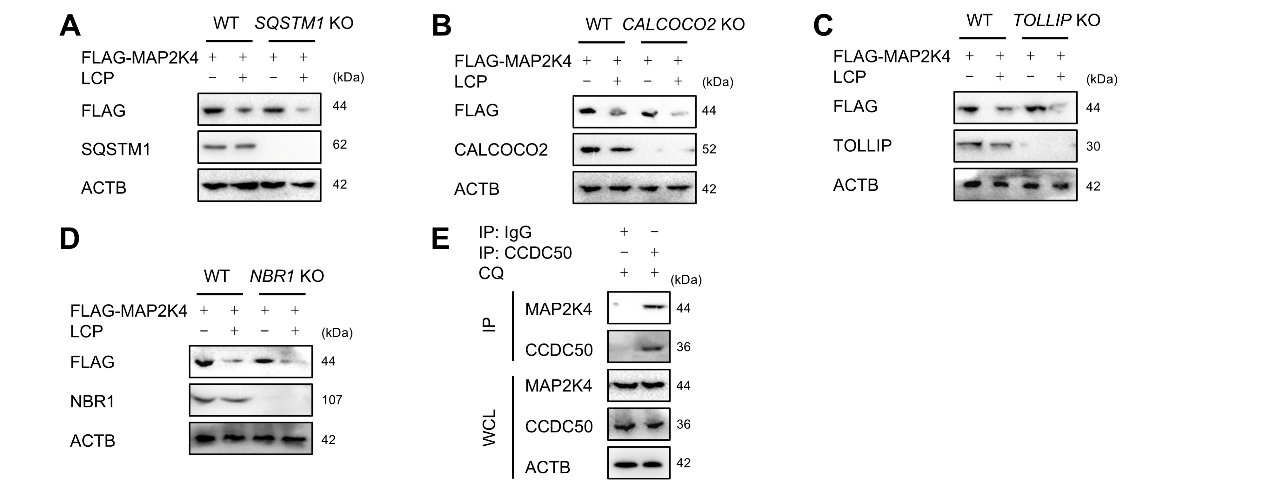


**Figure S5*.*** LCP mediates autophagic degradation of MAP2K4/MKK4 via the cargo receptor CCDC50. (**A-D**) WB analysis of FLAG-MKK4 expression in HEK293T cells with individual knockout (KO) of the genes encoding autophagy cargo receptors, including (**A**) *SQSTM1/p62*, (**B**) *CALCOCO2/NDP52*, (**C**) *TOLLIP*, and (**D**) *NBR1*, demonstrated that none of these receptors were required for LCP-induced MAP2K4/MKK4 degradation. (**E**) Co-IP experiments confirmed the endogenous interaction between CCDC50 and MAP2K4/MKK4 in chondrocytes.


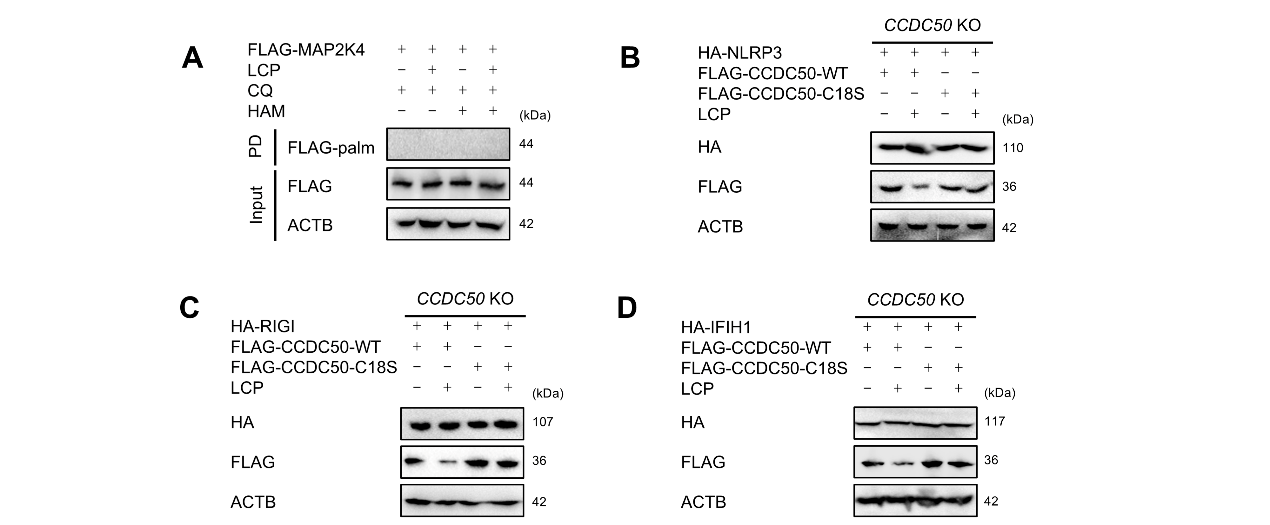


**Figure S6.** LCP enhances CCDC50 palmitoylation at Cys18 to promote MAP2K4/MKK4 degradation. (**A**) ABE assays showed that LCP did not affect palmitoylation levels of exogenous MAP2K4/MKK4 in HEK293T cells. (**B-D**) Mutation of Cys18 in CCDC50 did not impair the degradation of other known CCDC50 substrates, including (**B**) NLRP3, (**C**) RIGI, and (**D**) IFIH1/MDA5.


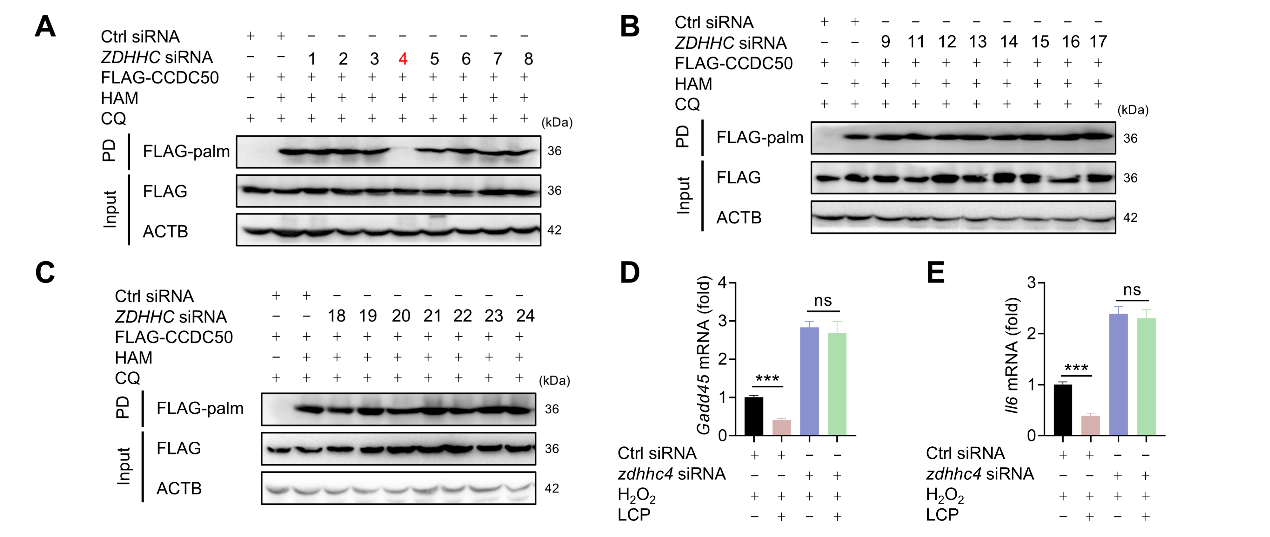


**Figure S7.** LCP inhibits chondrocyte senescence by targeting ZDHHC4 to catalyze the CCDC50 palmitoylation. (**A-C**) Palmitoylation (palm) screening of the *ZDHHC* family members identified *ZDHHC4* as essential for CCDC50 palmitoylation, with *ZDHHC4* silencing significantly abolishing CCDC50 palmitoylation. (**D-E**) qRT-PCR analysis showed that z*dhhc4* silencing abolished the LCP-mediated downregulation of senescence-related genes (**D**) *Gadd45* and (**E**) *Il6*. Data are expressed as mean ± SD. *** p < 0.001. ns = not significant.


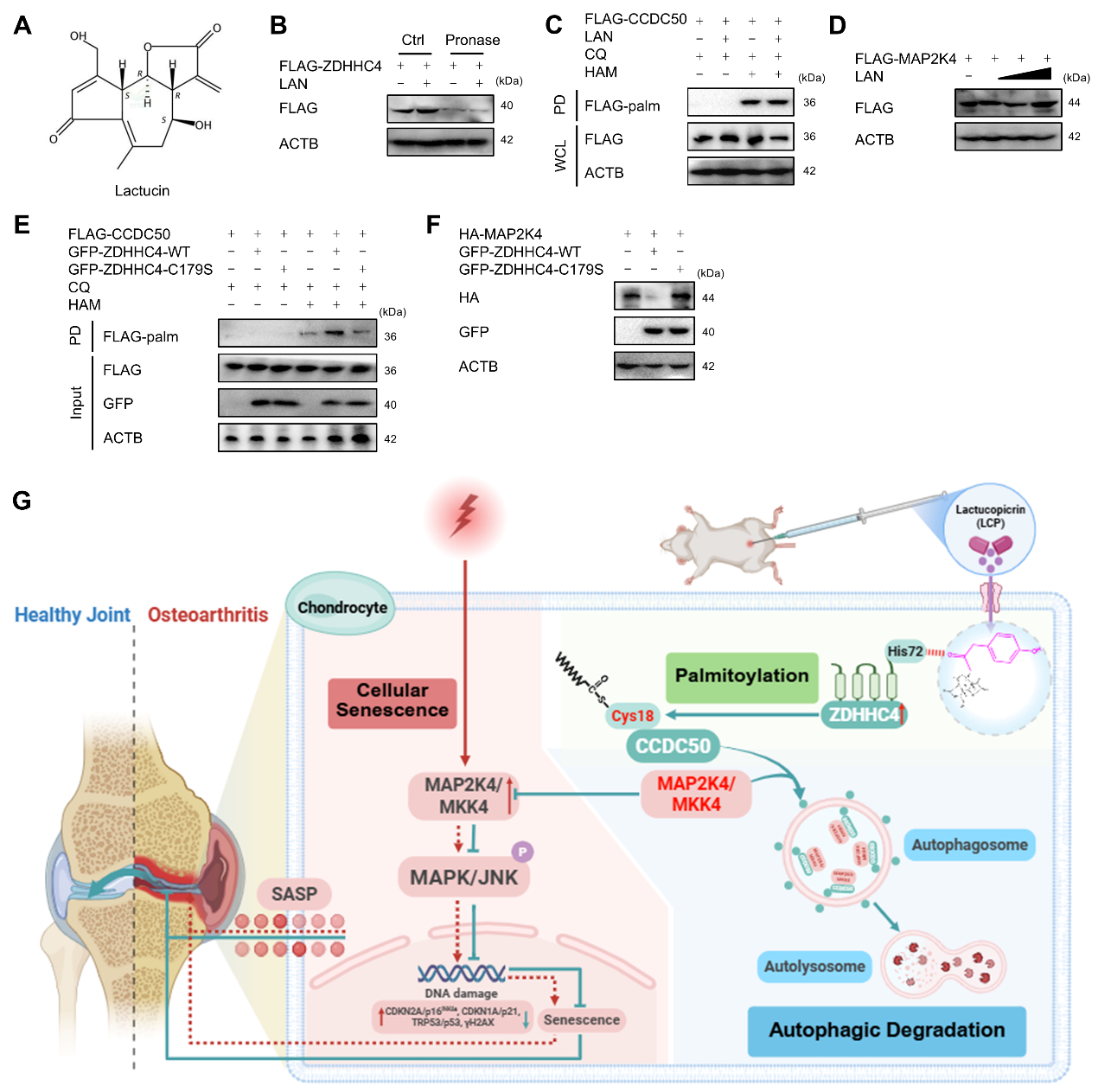


**Figure S8.** LCP binds to His72 of ZDHHC4 to enhance enzymatic activity and promote MAP2K4/MKK4 degradation*.* (**A**) The chemical structure of lactucin (LAN), a structural derivative of LCP lacking the p-hydroxybenzoic acid moiety. (**B**) DARTS assays demonstrated that LAN failed to stabilize ZDHHC4. (**C-D**) LAN treatment did not promote (**C**) CCDC50 palmitoylation or (**D**) MAP2K4/MKK4 degradation. (**E-F**) The enzymatic activity mutant of ZDHHC4 (C179S) disrupted (**E**) CCDC50 palmitoylation and (**F**) MAP2K4/MKK4 degradation in HEK293T cells. (**G**) Proposed mechanistic model illustrating how LCP alleviates OA progression in this study.
